# The RNA chaperone Hfq is essential for the virulence of *Salmonella typhimurium*

**DOI:** 10.1111/j.1365-2958.2006.05489.x

**Published:** 2007-01

**Authors:** Alexandra Sittka, Verena Pfeiffer, Karsten Tedin, Jörg Vogel

**Affiliations:** 1 Max Planck Institute for Infection Biology RNA Biology Group, Berlin, Germany; 2 Institut für Mikrobiologie und Tierseuchen, Freie Universität Berlin Berlin, Germany

## Abstract

The RNA chaperone, Hfq, plays a diverse role in bacterial physiology beyond its original role as a host factor required for replication of Q*β* RNA bacteriophage. In this study, we show that Hfq is involved in the expression and secretion of virulence factors in the facultative intracellular pathogen, *Salmonella typhimurium*. A *Salmonella hfq* deletion strain is highly attenuated in mice after both oral and intraperitoneal infection, and shows a severe defect in invasion of epithelial cells and a growth defect in both epithelial cells and macrophages *in vitro*. Surprisingly, we find that these phenotypes are largely independent of the previously reported requirement of Hfq for expression of the stationary phase sigma factor, RpoS. Our results implicate Hfq as a key regulator of multiple aspects of virulence including regulation of motility and outer membrane protein (OmpD) expression in addition to invasion and intracellular growth. These pleiotropic effects are suggested to involve a network of regulatory small non-coding RNAs, placing Hfq at the centre of post-transcriptional regulation of virulence gene expression in *Salmonella*. In addition, the *hfq* mutation appears to cause a chronic activation of the RpoE-mediated envelope stress response which is likely due to a misregulation of membrane protein expression.

## Introduction

The bacterial Sm-like protein, Hfq, has been increasingly recognized as a post-transcriptional regulator of global gene expression (Valentin-Hansen *et al*., 2004). Hfq was first identified in *Escherichia coli* as a host factor required for replication of Q*β* RNA bacteriophage (Franze de Fernandez *et al*., 1968), and shown to be an RNA-binding protein that forms homohexamers of ∼12 kDa subunits (Franze de Fernandez *et al*., 1972). Hfq was early observed to be an abundant protein (Carmichael *et al*., 1975), but its importance in uninfected bacteria remained unclear until it was shown that an *hfq* insertion mutant of *E. coli* exhibited broad, pleiotropic phenotypes affecting growth rate, cell morphology and tolerance of stress conditions (Tsui *et al*., 1994). Independently, genetic analysis of *Azorhizobium caulinodans* and *Yersinia enterocolitica* mutants, showing defects in nitrogen fixation or toxin production respectively, found that these phenotypes were due to mutations in *hfq* (Kaminski *et al*., 1994; Nakao *et al*., 1995). Subsequently, Hfq was shown to promote efficient translation of *rpoS* mRNA in *E. coli* and *Salmonella* (Brown and Elliott, 1996; Muffler *et al*., 1996), and to alter the stability of several other mRNAs (e.g. Vytvytska *et al*., 1998; Hajnsdorf and Regnier, 2000), indicating that this protein acts to regulate gene expression at the post-transcriptional level. Hfq has also emerged as a key player in mRNA translational control by small non-coding RNAs (sRNAs). Here, Hfq was first observed to be involved in translational repression of *rpoS* mRNA by OxyS, a small regulatory RNA that is part of the oxidative stress response in *E. coli* (Zhang *et al*., 1998). Since then, numerous *E. coli* sRNAs have been shown to associate with Hfq and to require this protein for their own stability and/or for interactions with their target mRNAs (reviewed in Valentin-Hansen *et al*., 2004; Majdalani *et al*., 2005; Romby *et al*., 2006). These include two *E. coli* sRNAs, DsrA and RprA, which activate *rpoS* translation in response to stress conditions (reviewed in Repoila *et al*., 2003); note, however, that the RpoS regulatory function of these sRNAs may not be conserved in *Salmonella* (Jones *et al*., 2006).

Several recent studies addressed a potential role of Hfq in the virulence of pathogenic bacteria. A *Brucella abortus hfq* mutant displayed significantly reduced survival in cultured murine macrophages, and attenuated virulence in a mouse model (Robertson and Roop, 1999). Similarly, Hfq was reported to be essential for the virulence of *Vibrio cholerae* (Ding *et al*., 2004). An *hfq* mutant of this bacterium fails to colonize the suckling mouse intestine, a model of cholera pathogenesis. Hfq also contributes to the pathogenesis of *Listeria monocytogenes* in mice (Christiansen *et al*., 2004), and to *Legionella pneumophila* virulence in amoeba and macrophage infection models (McNealy *et al*., 2005). Furthermore, the *hfq* mutation reduces the virulence of the opportunistic human pathogen *Pseudomonas aeruginosa* by affecting both cell-associated (flagellum, adhesion factors) as well as extracellular virulence factors, e.g. elastases and pyocyanin (Sonnleitner *et al*., 2003). In most of these cases, the observed virulence defects were accompanied by reduced stress tolerance, likely reflecting a compromised ability to cope with the harsh environment in the host cell (Robertson and Roop, 1999; Christiansen *et al*., 2004; McNealy *et al*., 2005).

A role for Hfq in bacterial virulence was first indicated by its requirement for efficient expression of the major stress sigma factor, σ^S^ (also known as RpoS, KatF or σ^38^) in the enteric bacteria, *E. coli* and *Salmonella*. Here, *hfq* mutants display greatly reduced RpoS levels in stationary phase, due to inefficient translation of the *rpoS* mRNA (Brown and Elliott, 1996; Muffler *et al*., 1996). In *Salmonella*, σ^S^ is an important virulence factor as it mediates the expression of the *Salmonella* plasmid virulence (*spv*) genes, which are required for systemic infection, and enables bacteria to cope with diverse stresses (nutrient deprivation, oxidative and acid stress, DNA damage) relevant to the environments faced in their mammalian hosts (Fang *et al*., 1992; Bang *et al*., 2005). A *Salmonella rpoS* mutant exhibits significantly reduced virulence in mice (Fang *et al*., 1992), and mutated *rpoS* alleles are often found in attenuated *Salmonella* strains (Robbe-Saule *et al*., 1995; Wilmes-Riesenberg *et al*., 1997).

Based on the importance of Hfq for σ^S^ expression and the many phenotypes shared by *hfq* and *rpoS* mutants in *E. coli* and *Salmonella* (Fang *et al*. 1992; Muffler *et al*., 1997), it has generally been assumed that Hfq would be important for *Salmonella* virulence. However, experimental evidence for a more general role of Hfq, i.e. beyond promoting *rpoS* mRNA translation, has so far been lacking. To address these questions, we constructed and characterized a set of *hfq* mutants and control strains in *Salmonella enterica* serovar Typhimurium (*S. typhimurium*). We find that loss of Hfq results in drastically reduced virulence *in vitro* and *in vivo*. These phenotypes, which are largely σ^S^-independent, are associated with loss of cell motility, altered membrane composition, reduced adhesion and abrogated effector protein secretion. The results indicate that Hfq plays a much more dominant role in *Salmonella* virulence than previously believed.

## Results

### Construction of *Salmonella hfq* mutant and control strains

**Fig. 1:**
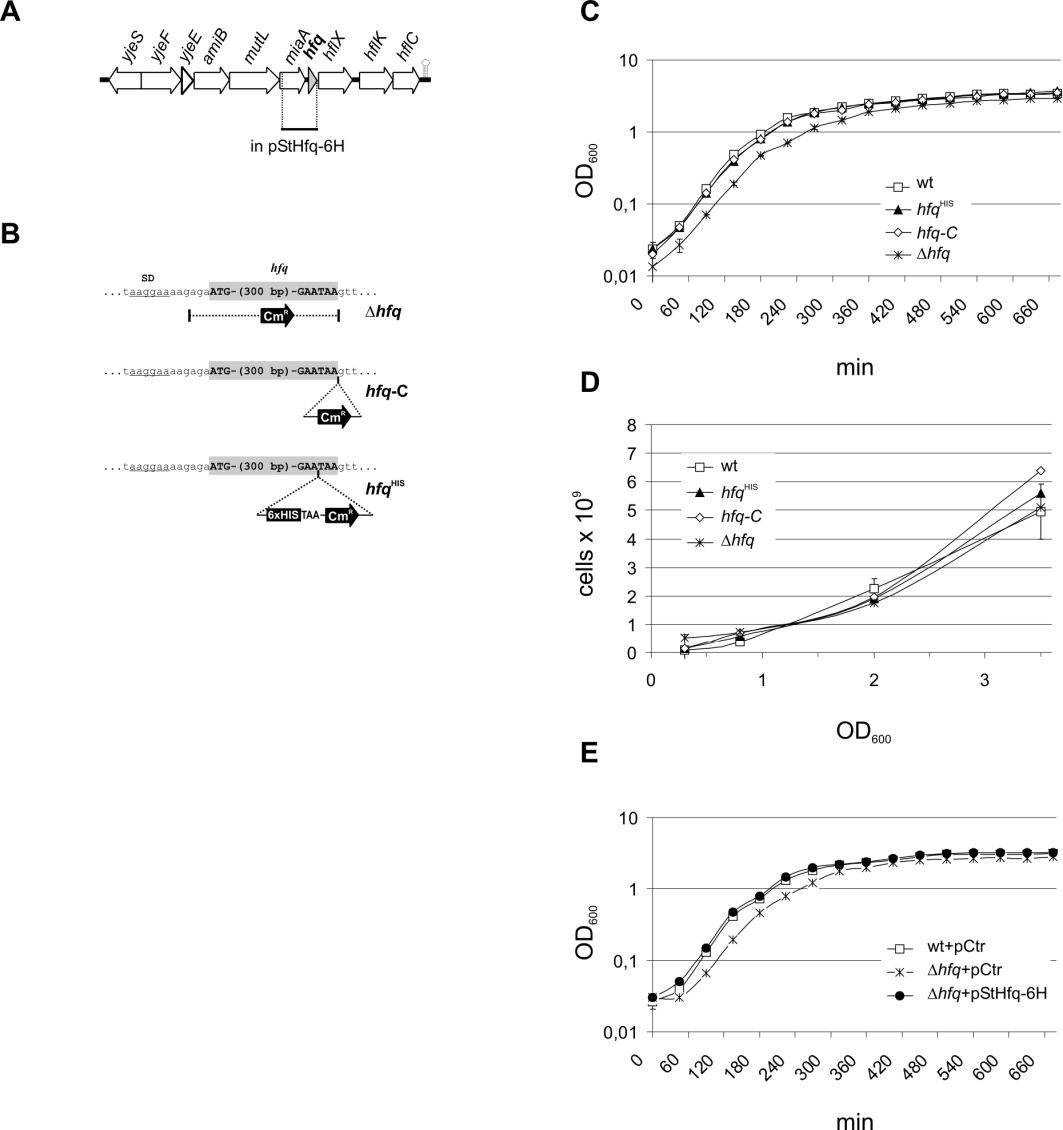
Details of *Salmonella hfq* mutants and their growth characteristics. A. Genomic location of *hfq* in SL1344. The region cloned on complementation plasmid, pStHfq-6H, is indicated. B. Schematic representation of the insertion sites of the *cat* resistance cassette in the deletion mutant Δ*hfq*, the control strain *hfq*-C, and the chromosomally HIS-tagged strain, *hfq*^HIS^. C and D. Growth and cell viability of *hfq* mutant strains (open squares: wild-type; filled triangles: *hfq*^HIS^; open diamonds: *hfq*-C; stars: Δ*hfq*). (C) OD_600_ values of triplicate cultures in LB medium were determined in 45 min intervals. (D) Bacteria were plated to determine viable counts (from triplicate cultures) at an OD of 0.3 and of 2, and 6 h after cultures had reached an OD of 2. E. Complementation of the slight growth defect of the Δ*hfq* strain by plasmid pStHfq-6H (open squares: wild-type strain carrying control plasmid pVP012; stars: Δ*hfq* carrying a control plasmid; filled circles: Δ*hfq* complemented with pStHfq-6H).

The *hfq* gene is located in clockwise orientation at bps 4604575–4604883 in the genome of *S. typhimurium* strain LT2 (McClelland *et al*., 2001). As in *E. coli*, it is located in the *yjeF-yjeE-amiB-mutL-miaA-hfq-hflX-hflK-hflC* cluster of genes (Fig. 1A), part of which may form an operon (Tsui and Winkler, 1994). The *Salmonella* and *E. coli hfq* genes are 93% and 94% identical at the nucleotide and amino acid level respectively, with all amino acid deviations being located in the Hfq C-terminal region (Brown and Elliott, 1996). The sequence of the *hfq* region taken from the unfinished genome of the virulent *Salmonella* strain used in this study, SL1344 (http://www.sanger.ac.uk/Projects/Salmonella), was compared with that of strain LT2 and found to be identical.

Based on the sequence data, three *hfq* mutant or control strains were constructed in SL1344 to study Hfq functions *in vivo* (Fig. 1B). In the Δ*hfq* mutant, the entire *hfq* coding region is replaced by a *cat* (chloramphenicol resistance) marker. As the *cat* gene used here does not carry a transcriptional terminator, transcription of the polycistron should be unaffected. *hfq*-C is a control strain in which the *cat* gene is inserted after the *hfq* stop codon. In control strain *hfq*^HIS^, the *cat* gene is inserted before the UAA stop codon. In addition, this latter insertion adds six histidine codons to the last *hfq* codon, thus producing a chromosomally encoded His-tagged Hfq protein.

### Growth characteristics of the *hfq* mutant and control strains

All three *hfq* strains formed normal colonies when grown on standard Luria–Bertani (LB) plates at 37°C, although the Δ*hfq* strain exhibited slightly slower growth. At room temperature (22°C) however, the Δ*hfq* mutant grew much more slowly than the wild type, seen as a smaller colony size, whereas the *hfq*-C and *hfq*^HIS^ derivatives showed normal growth (data not shown). When we compared the growth of all strains in LB liquid medium with aeration at 37°C, no differences were observed among the wild type, and the two control strains, *hfq*-C and *hfq*^HIS^ (Fig. 1C). The deletion mutant, Δ*hfq*, showed a longer lag phase after inoculation into fresh medium and reached stationary phase at a lower optical density as compared with the other three strains. However, parallel determination of viable counts at three different growth phases showed that cell viability of Δ*hfq* was uncompromised (Fig. 1D).

The observation that the *hfq*-C and *hfq*^HIS^ strains showed growth rates identical to the wild-type strain supported the suggestion that the slightly altered growth of the Δ*hfq* mutant was due to the lack of Hfq protein rather than to polar effects caused by the insertion of the *cat* cassette. To corroborate this, the *hfq*^HIS^ allele including 1014 bp of the upstream *miaA* coding sequence was cloned in a low-copy vector (pSC101* origin), resulting in plasmid pStHfq-6H. This plasmid fully complemented the reduced growth of the Δ*hfq* strain (Fig. 1E), also indicating that the major *hfq* promoter is located within the *miaA* coding region.

Certain growth conditions, e.g. oxygen limitation and high osmolarity, are known to activate *Salmonella* invasion gene expression *in vitro* (e.g. Lee and Falkow, 1990; Song *et al*., 2004). As these so-called *Salmonella* pathogenicity island 1 (SPI1)-inducing conditions were used extensively in this study (see below), we also determined the growth behaviour of all aforementioned strains under these conditions. As seen with aerobic growth, the Δ*hfq* mutant strain exhibited a slightly extended lag phase but reached the same optical density as the wild type while the two control strains *hfq*^HIS^ and *hfq*-C show growth indistinguishable from the wild-type strain (Fig. S1).

### The *hfq* mutation attenuates virulence in mice

**Fig. 2:**
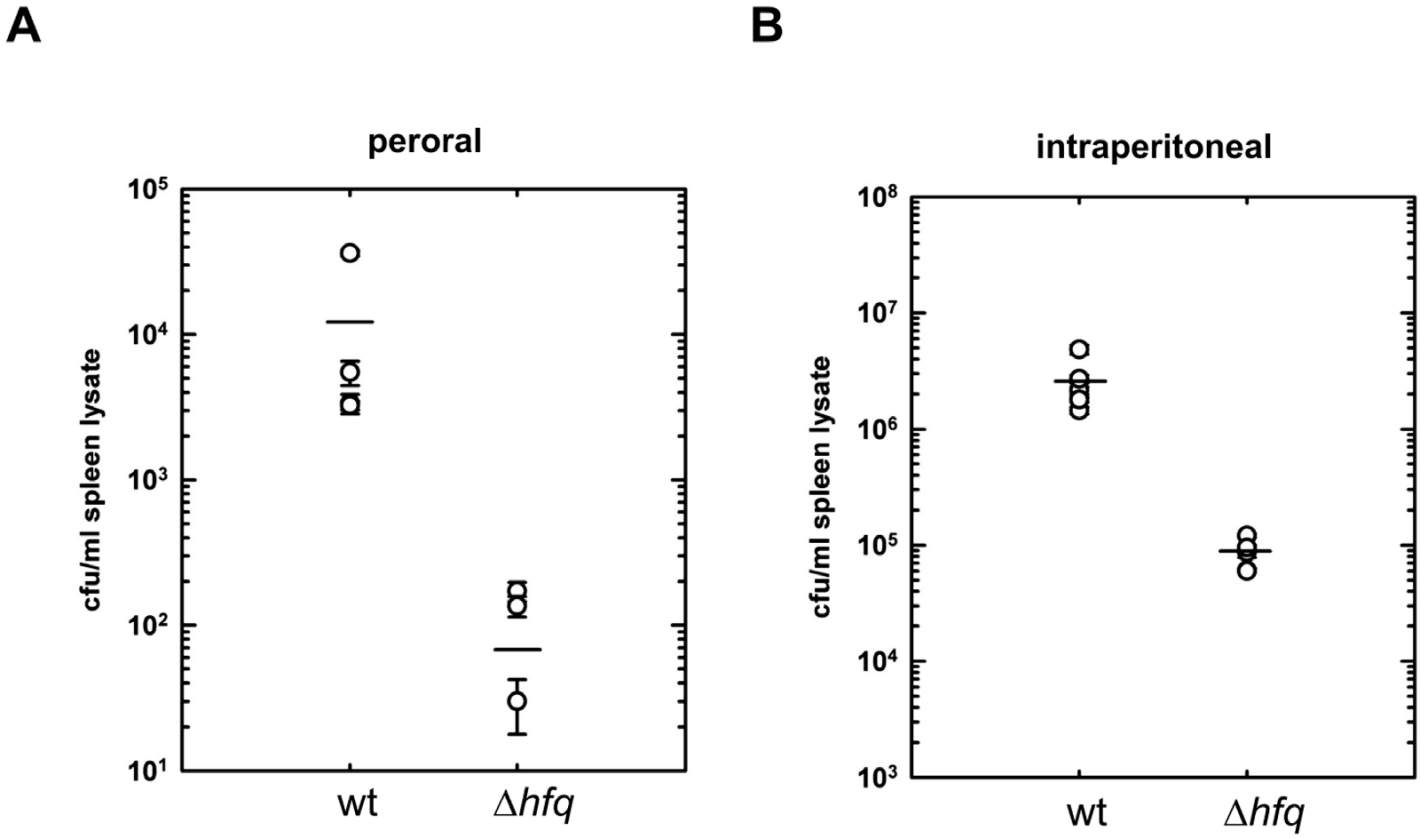
The *Δhfq* mutant is severely attenuated in mice. A. Groups of five Balb/c mice were infected perorally with suspensions of ∼10^8^ bacteria of either the wild-type or Δ*hfq* strains. Bacterial loads in spleen homogenates were determined 72 h post infection. For intraperitoneal infections (B) 1:1 mixtures of both, wild-type and Δ*hfq* strain, each strain at ∼10^5^ bacteria, were used for infections. Forty-eight hours post infection, spleens were removed and the cfu ml^−1^ for each strain was determined in spleen homogenates by plating to selective plates for calculation of the relative ratios of the two, co-infecting strains (competitive index, CI, see text).

To address the role of Hfq in *Salmonella* pathogenesis, we first examined the effect of the *hfq* deletion in a typhoid fever mouse model of *Salmonella* infection. Groups of 4- to 5-week-old, female Balb/c mice (five mice per strain) were infected perorally with 10^8^ cfu of either the wild-type or Δ*hfq* strains. Mice infected with the wild-type strain showed typical symptoms of infection beginning the following day, whereas mice infected with the Δ*hfq* mutant showed no signs of illness during the course of the experiment. The infected animals were sacrificed 72 h post infection, and organ colonization was determined by plating dilutions of homogenized spleen lysates to agar plates. As shown in Fig. 2A, the *hfq* mutant was recovered at > 100-fold reduced levels relative to the wild-type strain after peroral infection, and for at least two of the mice, no bacteria were recovered. These observations suggested that the *hfq* mutation resulted in defects in either invasion of intestinal epithelial cells, macrophage survival, or both.

To determine whether the virulence defect of the *hfq* mutant extended beyond invasion-related defects, mice were also co-infected intraperitoneally with a mixture of the wild-type and Δ*hfq* strains, where uptake by resident macrophages should circumvent the need for invasion. Two, independent experiments indicated that the *hfq* mutant showed at least a 30- to 100-fold reduced uptake and/or survival in macrophages and subsequent carriage to the spleen compared with the wild-type strain (Fig. 2B), leading to calculated competitive indices (CI; Shea *et al*., 1999) of 0.01–0.03. This is consistent with the idea that both uptake and intracellular survival/proliferation in macrophages were affected. It should be noted that the post-infection time points for determination of bacterial counts shown were chosen to avoid premature death of the infected animals. In preliminary experiments, in animals still surviving 1 week post infection in the mixed infection experiments, the Δ*hfq* strain showed a > 1000-fold reduction in cfu relative to the wild-type strain (CI of 0.0005−0.001; data not shown).

### The *hfq* mutant is impaired in the invasion of non-phagocytic cells

Oral infection by *Salmonella* results in active invasion of non-phagocytic epithelial cells of the host intestine. To determine the effect of the *hfq* mutation on the invasion rate of non-phagocytic cells *in vitro*, cultured HeLa cells were infected with the wild-type and several *hfq* mutant and control strain strains. A *Salmonella* SL1344 Δ*spi1* mutant, which lacks the entire SPI1, served as a negative control in these experiments. SPI1 encodes a type three secretion system (TTSS) and several effector proteins that mediate the uptake of *Salmonella* by non-phagocytic eukaryotic cells (Galan and Curtiss, 1989; Mills *et al*., 1995; Collazo and Galan, 1996). HeLa cells were infected with a multiplicity of infection (moi) of 10 with bacteria grown aerobically to early stationary phase (OD_600_ of 2). Following gentamicin treatment to kill remaining extracellular bacteria, the number of intracellular bacteria was determined 2 and 6 h post infection (Table 1).

**Table 1:** Invasion and intracellular replication (% of the bacterial input).

	Aerobic growth to early stationary phase (OD_600_ of 2), gentamicin protection assay (HeLa cells)		SPI1-inducing growth conditions, gentamicin protection assay (HeLa cells)		Aerobic growth to early stationary phase (OD_600_ of 2), macrophage survival assay (RawB)		
			
Strain/infection time	2 h	6 h	2 h	6 h	1 h	4 h	24 h
wt	14.16	30.38	29.4	82.11	16.53	29.79	47.48
*hfq*^HIS^	7.92	14.53	13.59	41.58	5.54	10.31	18.08
*hfq*-C	4.76	19.66	15.74	38.59	4.90	12.37	15.74
*Δhfq*	0.13	0.13	3.25	6.58	0.39	0.40	3.25
*Δspi1*	0.00	0.01	0.05	0.07	ND	ND	ND
*ΔrpoS*	8.79	22.61	22.88	65.89	ND	ND	ND
wt + pCtr	ND	ND	22.19	70.74	9.98	26.55	28.63
*Δhfq* + pCtr	ND	ND	2.35	6.29	0.54	0.57	0.67
*Δhfq* + pStHfq-6H	ND	ND	40.87	118.38	15.16	41.21	42.24

The *hfq* deletion mutant showed a 100-fold reduced initial rate of invasion at 2 h post infection compared with the wild-type strain. We also compared the number of intracellular bacteria present after an additional 4 h. Within these 4 h, the number of wild-type bacteria doubled, whereas the number of *hfq* mutant bacteria remained unchanged, suggesting an intracellular growth defect in addition to an invasion defect. Despite its drastic invasion defect, the invasion rate of the *hfq* mutant remains above that of a non-invasive Δ*spi1* mutant for which only single cells could be recovered (Table 1 and Fig. S2A).

To determine whether the *hfq* mutant was still impaired in invasion when grown under SPI1-inducing conditions, the invasion assays were repeated with bacterial cultures grown for 12 h under high-salt, oxygen-limiting conditions (Table 1 and Fig. S2B). These growth conditions increased the invasion rate of both the wild type and the Δ*hfq* strain to 30% and 3% respectively (as calculated for the 2 h time point). However, the Δ*hfq* strain remained 10-fold less invasive than the wild type, and intermediate with respect to the non-invasive Δ*spi1* mutant. While the wild-type strain showed more than one replication in additional 4 h, the Δ*hfq* strain only doubled in the 4 h period.

Three other strains included as controls in all of these experiments, Δ*rpoS*, *hfq*-C and *hfq*^HIS^, all displayed only slightly reduced invasion rates in the range of 1.3- to threefold in comparison with the wild type, and none of these strains were affected in intracellular growth (Table 1 and Fig. S2A and B). To corroborate that the lack of Hfq protein was the main cause of the invasion defect of the Δ*hfq* mutant, we tested whether it could be complemented by a plasmid-borne *hfq* allele. Providing Hfq *in trans* with plasmid pStHfq-6H not only fully restored invasion to the *hfq* deletion strain, but enhanced invasion relative to the wild type (Table 1 and Fig. S2B). Taken together, these data suggest that Hfq is required for efficient invasion of non-phagocytic cells, which is likely to underlie the strong attenuation of virulence seen in oral mouse infections.

We also examined both the invasion and long-term intracellular growth phenotypes of the Δ*hfq* mutant in an intestinal epithelial cell line (Fig. S3A). Consistent with the results using the HeLa cell line, the initial invasion rate of LoVo cells was 10- to 100-fold reduced at either a 10-fold higher or equivalent infective dose as the wild-type strain respectively. In addition, whereas the wild-type strain showed an approximately 10- to 20-fold increase in intracellular cfu over a 24 h period, the Δ*hfq* strain showed either no change or a slight reduction in viable bacteria over the same period. These results were consistent with a requirement for Hfq for both invasion as well as intracellular replication in non-phagocytic cells.

### The *hfq* strain survives but shows an intracellular growth defect in macrophages

*Salmonella* survival in the host is also dependent on the ability to survive and replicate in macrophages. To test a possible role for Hfq in macrophage survival, we infected *in vitro* cultured murine macrophages (RawB) with equal numbers of wild-type and *hfq* mutant bacteria (Table 1 and Fig. S2C). At 1 h post infection, we noted 30-fold fewer intracellular bacteria in macrophages infected with the *hfq* mutant, likely reflecting the reduced invasion rate of this strain. However, complementation with plasmid pStHfq-6H fully restored macrophage invasion, comparable to levels observed with wild-type bacteria. Intracellular replication as determined 4 and 24 h after infection also revealed drastic differences between the wild-type strain and the *hfq* deletion mutant. While the wild-type and the *hfq*-C and *hfq*^HIS^ control strains at least doubled within the 4 h post infection, the *hfq* deletion mutant showed no significant increase in intracellular bacteria per macrophage. At 24 h post infection the number of intracellular bacteria had increased to > threefold as compared with the 1 h time point for the wild-type, the control strains and the complemented deletion mutant (Table 1).

In other experiments, infection of the J774A.1 murine macrophage cell line showed a similar reduction in initial uptake, but no significant increase in intracellular cfu for up to 24 h (Fig. S3B). Thus, Hfq appeared to have little or no effect on the expression of genes required for macrophage survival, although the lack of significant intracellular growth in both epithelial and macrophage cell lines suggested an effect on expression of the second, major pathogenicity island, SPI2, which is required for intracellular proliferation (Shea *et al*., 1996; Cirillo *et al*., 1998; Hensel *et al*., 1998).

### Lack of Hfq results in global changes of protein expression and loss of protein secretion

**Fig. 3:**
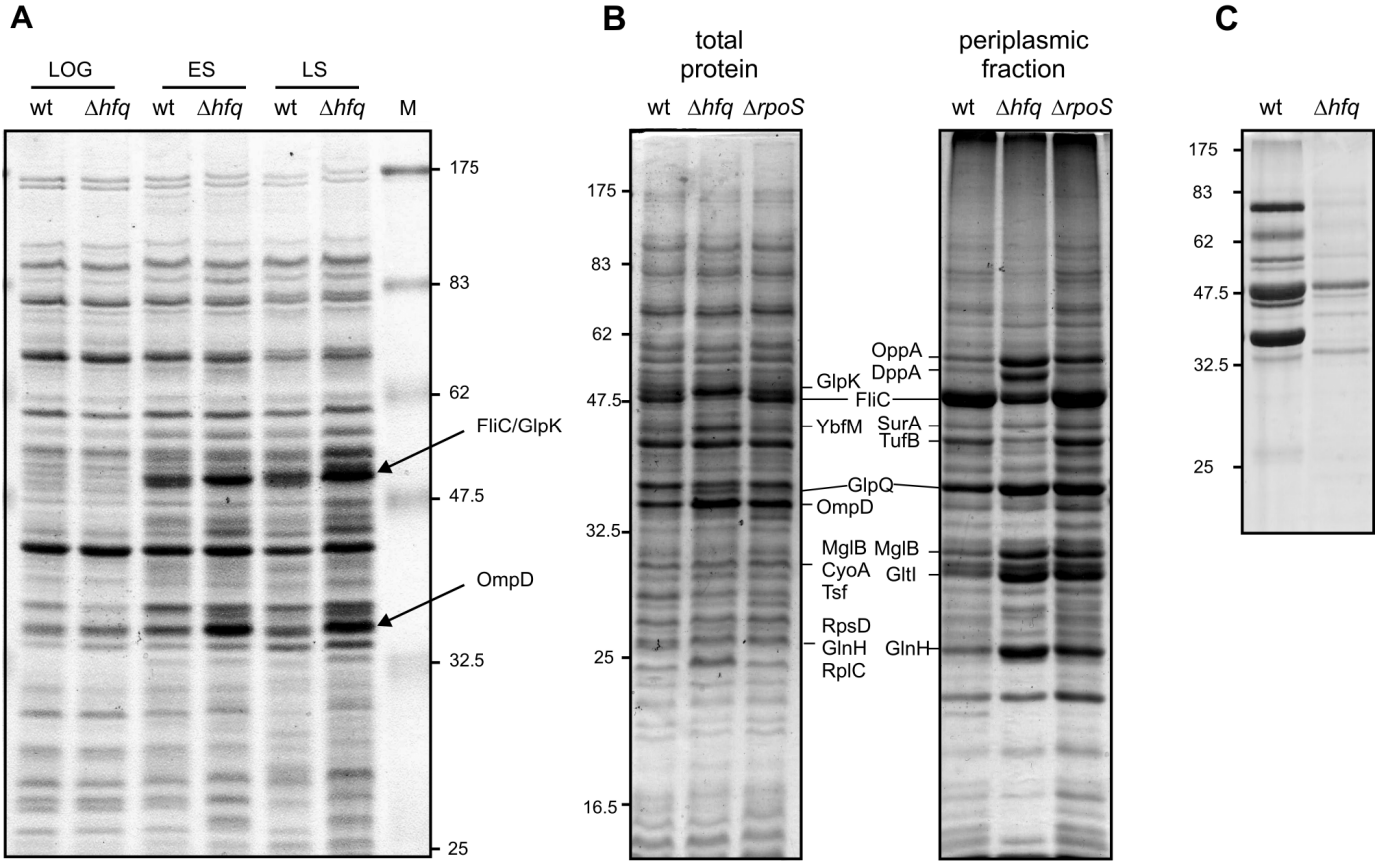
Altered protein expression in *Salmonella Δhfq*. SDS-PAGE (10–12% gels) of protein samples of SL1344 wild-type and Δ*hfq* prepared from different growth phases (LOG: logarithmic phase, OD_600_ of 0.3; ES: early stationary phase, OD_600_ of 2; LS: late stationary phase, 6 h after cells had reached an OD_600_ of 2). A. Total protein samples. B. Total protein and periplasmic fractions; samples of a Δ*rpoS* strain were included as an additional control. C. Secreted protein fractions of early stationary phase bacteria.

Considering the pleiotropic effect of Hfq on mRNA stability and translational regulation in other bacteria, we sought to determine Hfq-dependent changes in protein expression. We first compared the whole-cell protein patterns in one-dimensional gels of wild-type and Δ*hfq* cells from cultures grown aerobically in l-broth in three different growth phases: exponential growth, early and late stationary phase. As shown in Fig. 3A, Δ*hfq* cells exhibit no significant difference to the wild type in exponential phase. In contrast, in stationary phase the Δ*hfq* mutation showed a markedly different protein pattern, with the most prominent and reproducible changes being two abundant protein bands of ∼40 and ∼55 kDa (Fig. 3A). Mass spectrometry (MALDI-TOF) identified the 40 kDa band as the major outer membrane protein (OMP), OmpD. Analysis of the 55 kDa band proved more complex, because MALDI-TOF analysis indicated the presence of two proteins, GlpK (glycerol kinase) and FliC (major phase-1 flagellin). This band was further resolved with longer gel runs (Fig. 3B, left panel) and revealed that in the Δ*hfq* mutant, FliC levels were strongly reduced whereas GlpK accumulated to higher levels. Parallel analysis of the protein profile of an *rpoS* deletion strain showed that the Hfq-dependent regulation of OmpD, FliC and GlpK, was not related to lower σ^S^ levels in Δ*hfq* cells. Additional analyses revealed an increase in the levels of HtrA, YbfM, OmpF, CyoA and Tsf, and a decrease of the ribosomal proteins RpsD and RplC in the Δ*hfq* strain*.* To obtain a preliminary picture of global changes in the expression profiles of less abundant proteins, we also analysed early stationary phase samples of wild-type and Δ*hfq* cells resolved on two-dimensional gels (Fig. S4). Of the 69 protein candidates analysed by MALDI-TOF, 32 were upregulated in Δ*hfq* cells, whereas 37 showed downregulation. These results are summarized in Table 2 (further details are given in Table S1).

**Table 2:** Results of 1D and 2D gel analysis of protein patterns of SL1344 wild-type and Δ*hfq* cultures grown to early stationary phase (OD_600_ = 2).

Candidate protein^a^	Regulation^b^	Localization^c^	Function^d^	Analysis^e^
CarA	–	CP	Carbamoyl-phosphate synthetase, glutamine-hydrolysing small subunit	2D
SurA	+	CP	Peptidyl-prolyl *cis*-*trans* isomerase, survival protein	1D, 2D
HtrA	+	PP	Periplasmic serine protease Do, heat shock protein	1D, 2D
PyrH	–	CP	Uridine 5′-monophosphate kinase	2D
Upp	–	CP	uracil phosphoribosyltransferase	2D
YaeT	+	(OM)	Putative outer membrane antigen	2D
GltI	+	PP	ABC transporter periplasmic binding protein; ABC superfamily, glutamate/aspartate transporter	1D, 2D
SucD	–	CP	Succinyl-CoA synthetase, alpha subunit	2D
Pal	+	PP	Tol protein required for outer membrane integrity, uptake of group A colicins, and translocation of phage DNA to cytoplasm	2D
YbgF	–	(PP)	Putative periplasmic protein	2D
Dps	–	CP	Stress response DNA-binding protein; starvation induced resistance to H_2_O_2_; DNA protection during starvation protein	2D
CspD	+	CP	Cold shock-like protein CspD; similar to CspA but not cold shock induced	2D
TrxB	–	CP	Thioredoxin reductase; thioredoxin reductase	2D
FabF	–	CP	3-oxoacyl-[acyl-carrier-protein] synthase II	2D
IcdA	+	CP	Isocitrate dehydrogenase in e14 prophage, specific for NADP+	2D
PagC	+	OM	PhoP regulated: reduced macrophage survival; virulence membrane protein PagC precursor	2D
STM1254	–	(OM)	Putative outer membrane lipoprotein	2D
STM1328	–	(OM)	Putative OMP	2D
AroD	–	CP	3-Dehydroquinate dehydratase	2D
LppB	–	OM	Putative methyl-accepting chemotaxis protein; major outer membrane lipoprotein	2D
LppA	–	OM	Murein lipoprotein, links outer and inner membranes; major outer membrane lipoprotein	2D
YnaF	–	CP	Putative universal stress protein	2D
Tpx	+	CP	Thiol peroxidase	2D
TrpB	–	CP	Tryptophan synthase beta chain	2D
OppA	+	PP	ABC superfamily, oligopeptide transport protein with chaperone properties	1D, 2D
KdsA	–	CP	3-deoxy-D-manno-octulosonic acid 8-P synthetase	2D
PrsA	–	CP	Phosphoribosylpyrophosphate synthetase	2D
FliC	–	OM/SUP	Flagellin, filament structural protein	2D
Gnd	–	CP	Gluconate 6-phosphate dehydrogenase, decarboxylating	2D
GlpQ	+	PP	Glycerophosphodiester phosphodiesterase, periplasmic	1D, 2D
AckA	–	CP	Acetate kinase A (propionate kinase 2)	2D
HisJ	–	PP	ABC superfamily, histidine-binding periplasmic protein	2D
CysP	+	PP	ABC superfamily, thiosulphate transport protein	2D
MaeB	+	CP	Paral putative transferase; phosphate acetyltransferase	2D
NlpB	+	OM	Lipoprotein-34	2D
STM2494	+	(IM)	Putative inner membrane or exported	2D
NifU	–	CP	NifU homologue involved in Fe-S cluster formation	2D
YfiA	–	CP	ribosome associated factor, stabilizes ribosomes against dissociation; putative sigma(54) modulation protein	2D
LuxS	–	CP	Quorum sensing protein, produces autoinducer – acyl-homoserine lactone-signalling molecules	2D
SipA	–	SUP	Cell invasion protein	2D
SipC	–	SUP	Cell invasion protein	2D
GudD	–	CP	d-Glucarate dehydratase	2D
Ptr	+	PP	Protease III	2D
OmpX	–/+	OM	Ail and ompX homologue; outer membrane protein X precursor	2D
YraP	+	(PP)	Paral putative periplasmic protein; possible lipoprotein	2D
RbfA	–	CP	Ribosome-binding factor, role in processing of 10S rRNA	2D
GreA	+	CP	Transcription elongation factor, cleaves 3′ nucleotide of paused mRNA	2D
Mdh	–/+	CP	Malate dehydrogenase	2D
AccB	+	CP	acetyl-CoA carboxylase, BCCP subunit, biotin carboxyl carrier protein	2D
FkpA	+	CP	FKBP-type peptidyl-prolyl *cis*-*trans* isomerase (rotamase)	2D
DppA	+	PP	ABC superfamily, dipeptide transport protein	1D, 2D
YiaD	+	(OM)	Putative outer membrane lipoprotein	2D
Kbl	–	CP	2-amino-3-ketobutyrate CoA ligase (glycine acetyltransferase)	2D
PstS	+	PP	ABC superfamily, high-affinity phosphate transporter	2D
RbsB	+	PP	ABC superfamily, d-ribose transport protein; d-ribose-binding periplasmic protein	2D
FadA	–	CP	3-ketoacyl-CoA thiolase (thiolase I, acetyl-CoA transferase), small (beta) subunit of the fatty acid-oxidizing multienzyme complex	2D
RplL	–	CP	50S ribosomal subunit protein L7/L12	2D
MalE	–	PP	ABC superfamily maltose transport protein, substrate recognition for transport and chemotaxis	2D
AphA	+	PP	Non-specific acid phosphatase/phosphotransferase, class B	2D
OsmY	–	PP	Hyperosmotically inducible periplasmic protein, RpoS-dependent stationary phase gene	2D
Tsf	+	CP	Protein chain elongation factor EF-Ts	1D
CyoA	+	IM	Cytochrome o ubiquinol oxidase subunit II	1D
YbfM	+	(OM)	Putative OMP	1D
GlnH	+	PP	ABC superfamily (bind_prot), glutamine high-affinity transporter	1D
OmpF	+	OM	OMP 1a (ia; b; f), porin	1D
MglB	+	PP	ABC superfamily (peri_perm), galactose transport protein	1D
STM2786	+	PP	Tricarboxylic transport	1D
RpsD	–	CP	30S ribosomal subunit protein S4	1D
RplC	–	CP	50S ribosomal subunit protein L3	1D
GlpK	+	CP	Glycerol kinase	1D
TufB	–	CP	Protein chain elongation factor EF-Tu (duplicate of tufA)	1D

a Nomenclature according to coliBASE (http://colibase.bham.ac.uk/; Chaudhuri *et al*., 2004).

b Up- or downregulation in *hfq* strain as compared with SL1344.

c Predicted subcellular protein localization: CP, cytoplasmic; PP, periplasmic; OM, outer membrane; IM, inner membrane; SUP, secreted.

d Functional classification according to KEGG (http://www.genome.jp/kegg/; Goto *et al*., 1997).

e Protein identified on one-dimensional (1D) or two-dimensional (2D) gel.

See Table S1 for further details.

Loss of Hfq also affected the composition of the periplasmic protein population (Fig. 3B, right panel). While some of the changes in protein expression seen in Δ*hfq* cells are shared with the *rpoS* deletion strain (e.g. OppA and GltI), loss of Hfq leads to a specific increase in DppA, a decrease in TufB levels, and higher levels of OppA, MglB, GltI and GlnH as compared with the Δ*rpoS* strain (Table 2).

**Fig. 5:**
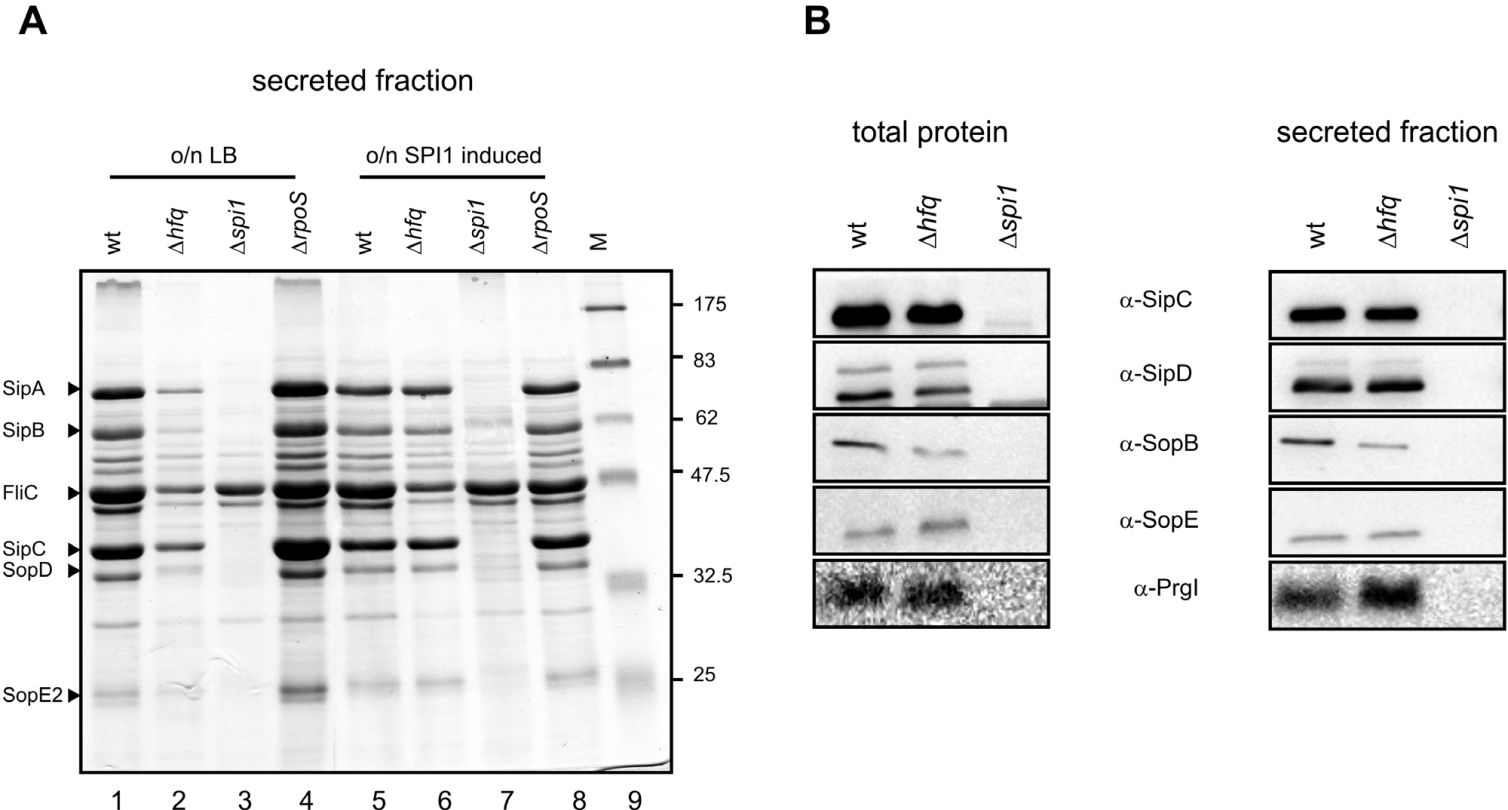
SPI1-inducing conditions restore effector levels and their secretion in the Δ*hfq* strain. A. Comparison of secreted proteins of wild-type, Δ*hfq*, Δ*spi1* and Δ*rpoS* grown for 12 h under standard conditions (lanes 1–4) or SPI1-inducing conditions (lanes 5–8) by SDS-PAGE analysis. B. Western blot detection of effector and needle proteins in total protein samples and secreted fractions of bacteria grown for 12 h under SPI1-inducing conditions. Bacterial strains from left to right: wild-type, Δ*hfq*, Δ*spi1*.

The most drastic effects of the *hfq* deletion, however, were observed with the secreted protein fraction (Fig. 3C). FliC, the most prominent protein found in *Salmonella* supernatants (Komoriya *et al*., 1999) and other secreted proteins typically seen in SL1344 supernatants, e.g. effector proteins that are translocated by the SPI1 TTSS (Ehrbar *et al*., 2002), were either strongly reduced or undetectable. The loss of secreted SPI1 effectors was consistent with the reduced invasion phenotype of the Δ*hfq* strain. None of these reductions were observed with the Δ*rpoS* strain (Fig. 5A).

### Overexpression of HilA in *Δhfq* rescues SPI1 effector protein expression but not secretion

Consequently, we sought to determine if the Hfq-dependent loss of secreted SPI1 effectors was due to a more general defect on SPI1 gene expression. The activation of SPI1 genes is mediated by a transcription factor cascade. On top of this cascade, the transcription factors, HilC and HilD, along with RtsA (encoded outside SPI1) cooperate to transmit environmental signals that lead to derepression of *hilA* (Bajaj *et al*., 1996; Lucas and Lee, 2001; Schechter and Lee, 2001; Ellermeier *et al*., 2005). HilA is the SPI1 major transcriptional activator responsible for most of the SPI1 TTSS and effector gene expression, both directly and indirectly through its activation of InvF (Darwin and Miller, 1999; Eichelberg and Galan, 1999; Lostroh and Lee, 2001). In addition, HilA also activates expression of secreted effector proteins encoded outside SPI1, e.g. SopB encoded within SPI5 (Ahmer *et al*., 1999).

**Fig. 4:**
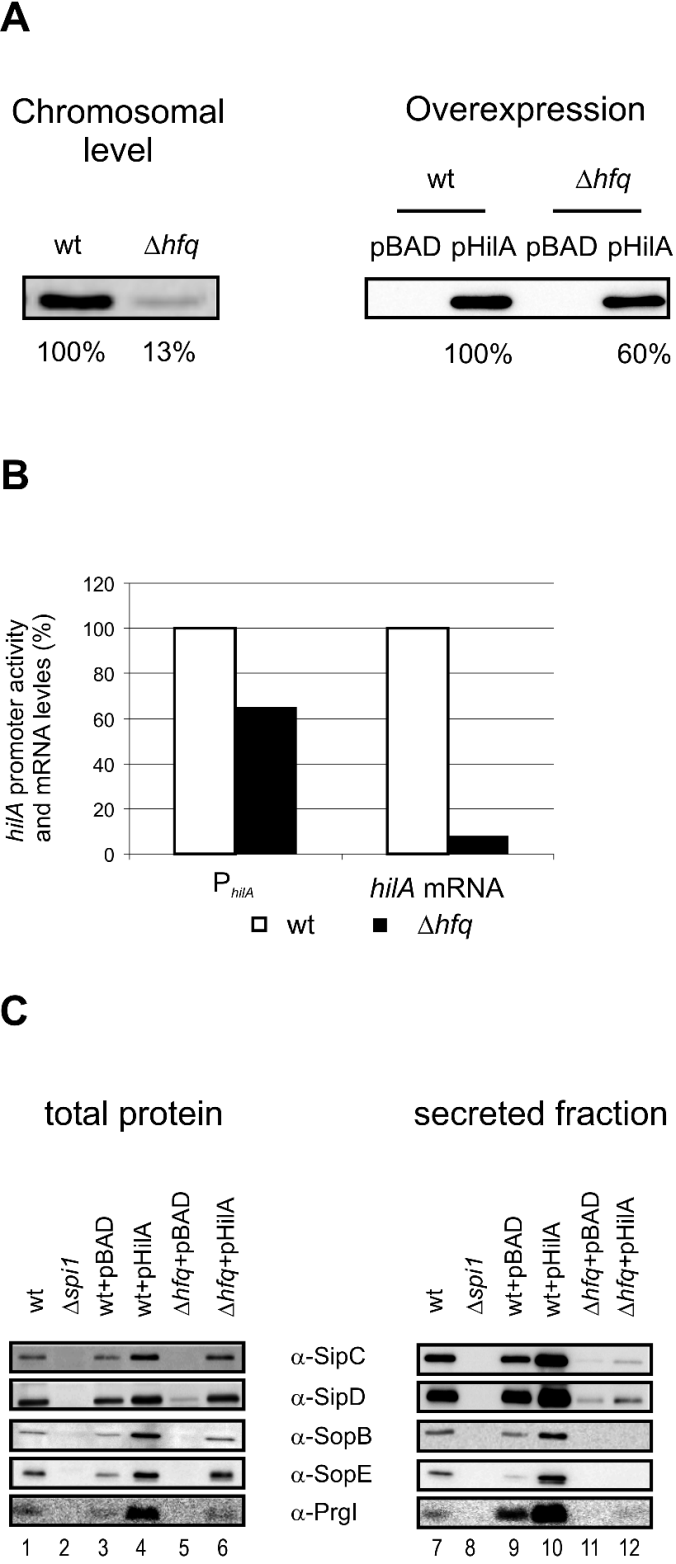
The *hfq* deletion mutant is impaired in HilA expression and shows reduced effector levels. A. HilA levels in wild-type and Δ*hfq Salmonella* grown to early stationary phase. Shown are Western blots probed for chromosomally encoded HilA^FLAG^ protein (left panel), or HilA^myc^ protein as expressed from pBAD-HilA expression plasmid (right panel). Bacteria carrying the empty pBAD vector were included as control. B. *hilA* promoter activity determined with a transcriptional *hilA-gfp* fusion in early stationary phase (P_*hilA*_), and *hilA* mRNA levels as determined by Northern analysis. Given are relative values obtained for Δ*hfq*, with the levels determined for the wild-type strain set to 100%. C. Western blot detection of effector and needle proteins in total protein samples and secreted fractions of bacteria grown to early stationary phase. Bacterial strains from left to right: wild-type, Δ*spi1,* wild-type strain carrying a pBAD control vector, wild-type strain carrying a pBAD-HilA expression plasmid, Δ*hfq* carrying a pBAD control vector, Δ*hfq* with pBAD-HilA expression plasmid. All strains were grown in LB medium complemented with 0.05% l-arabinose to facilitate HilA expression from plasmid pBAD-HilA.

To quantify the amount of HilA protein, we constructed a chromosomal FLAG epitope-tagged derivative of the *hilA* gene. Quantification of Western blot signals obtained for HilA^FLAG^ revealed a > sixfold reduction of the protein in the Δ*hfq* mutant as compared with the wild type (Fig. 4A, left panel). In addition, Northern blot quantification showed that in Δ*hfq* cells *hilA* mRNA was reduced to ∼8% of wild-type levels (Fig. 4B). Several transcriptional reporter fusions were also used to determine if the changes in *hilA* expression resulted from a reduced *hilA* promoter activity (Fig. 4B). Depending on the fusion used, *hilA* transcription in Δ*hfq* was found to be reduced to between 30% and 70% of wild-type levels. Collectively, this suggested that Hfq regulates HilA synthesis at both the transcriptional and the post-transcriptional level.

To verify that the lower HilA levels in the Δ*hfq* strain cause a reduction of SPI1 effector protein synthesis, we first determined the intracellular levels of SipC, SipD, SopB and SopE on Western blots, all of which were readily detected in wild-type cells (Fig. 4C, lanes 1 and 3). In stark contrast, no (SipC, SopB, SopE) or drastically reduced (SipD) signals were obtained in the Δ*hfq* background (lane 5). To determine whether HilA overexpression could restore effector protein expression in the absence of Hfq, the wild-type and the Δ*hfq* strains were transformed with plasmid pBAD-HilA (Lostroh *et al*., 2000), which carries a *myc*-tagged *hilA* gene under control of an arabinose-inducible P_BAD_ promoter. Arabinose induction yielded comparable HilA^myc^ protein levels in both genetic backgrounds (Fig. 4A, right panel), and fully restored the intracellular levels of effector proteins in Δ*hfq* cells to wild-type amounts (Fig. 4C, compare lanes 3 and 6). We next examined whether HilA overexpression could also restore effector protein secretion. Supernatants of the same cultures used for whole-protein determinations in Fig. 4C were examined for extracellular levels of the aforementioned effector proteins. In stark contrast to the full restoration of intracellular effector protein levels, HilA expression failed to significantly increase the extracellular amounts of these proteins in the Δ*hfq* strain (lanes 11 and 12). HilA overexpression in the Δ*hfq* background was therefore able to overcome the loss of expression of these effector proteins but not of their secretion.

One possible explanation for this secretion defect was that the *hfq* mutation does not permit assembly of a functional SPI1 secretion apparatus. The secreted PrgI protein, the main component of the needle of the SPI1-encoded TTSS, provides a testable marker for a functional secretion apparatus (Kimbrough and Miller, 2000; Kubori *et al*., 2000). We determined both the intra- and extracellular PrgI levels in all of the strains, and found that this protein was absent in the Δ*hfq* mutant (Fig. 4C, lower panel, lanes 1 and 3 versus 5, lanes 7 and 9 versus 11). In contrast, HilA overexpression led to elevated intracellular and secreted PrgI levels in the wild-type but not *hfq* strains (lanes 4 and 6 versus 10 and 12). These results indicated that under aerobic growth conditions, Hfq affected SPI1 expression at multiple levels, and was required for the expression of the TTSS structural genes independent of HilA expression.

### Effector protein secretion independent of *hfq* under SPI1-inducing conditions

As we had observed that the invasion defect of the Δ*hfq* strain was less pronounced when grown under SPI1-inducing conditions, we considered whether this was the result of improved effector protein secretion. Indeed, supernatants of Δ*hfq* cells cultured under SPI1-inducing conditions displayed a protein pattern close to the wild type (Fig. 5A, compare lanes 5 and 6), except for the flagellar protein, FliC. When these samples were probed on Western blots for the effectors SipC, SipD, SopB and SopE, a similar level of secretion as for the wild-type strain was evident for the *hfq* mutant (Fig. 5B). Furthermore, under these growth conditions, the Δ*hfq* strain accumulated the needle protein, PrgI, to wild-type levels both intracellularly and in the supernatant, arguing that under this growth condition, Δ*hfq* bacteria also possess a fully active SPI1 TTSS.

### Impaired adhesion contributes to the non-invasive phenotype of *Δhfq*

Although the Δ*hfq* strain appeared to show wild-type levels of expression in terms of SPI1 function when grown under SPI1-inducing conditions, it was puzzling that the mutant remained much less invasive. One important factor that contributes to *Salmonella* invasion of host cells in addition to SPI1 function is successful adhesion to epithelial cells, mediated by fimbrial adhesins. We therefore performed assays to compare the adhesion phenotypes of the wild-type and Δ*hfq* strains. To better visualize bacteria, both strains were transformed with a low-copy plasmid that constitutively expresses green fluorescent protein (GFP). Transformants were grown under SPI1-inducing conditions, and used for infection of HeLa cells at a moi of 50. Following incubation at 37°C for 1 h, bacteria that had not attached to the HeLa cells were removed by extensive washing of the cells. The remaining bacteria and cells were fixed, and the number of bacteria per HeLa cell determined by fluorescence microscopy (Fig. S5A). For the wild-type strain, an average of ∼30 bacteria per HeLa cell were found to be adherent. In contrast, the average number observed with the Δ*hfq* strain was significantly lower, i.e. ∼10 bacteria per HeLa cell. For both strains, we observed that a significant proportion of bacteria became internalized during the 1 h incubation step prior to counting. As the assay does not allow us to clearly distinguish extra- from intracellular bacteria, our calculation includes all bacteria associated with HeLa cells, based on the assumption that every internalization event was preceded by successful adhesion.

To better separate adhesion from invasion rates, bacterial adherence was also determined in HeLa cell infection assays without gentamicin treatment. To this end, serial dilutions of HeLa cells and adhered bacteria were plated on LB agar 30 min upon infection, and cfu determined (Fig. S5B). These experiments revealed a > twofold reduction in adhesion of the *hfq* deletion mutant as compared with wild-type *Salmonella* (25% adherence of wild-type compared with 11% of the *hfq* strain related to the input). In contrast, adherence of the two control strains, *hfq*^HIS^ and *hfq*-C, did not significantly differ from the wild type (21% and 24% respectively). Collectively, the data suggest that a lower adhesion rate may contribute to the non-invasive phenotype of the *hfq* strain.

### *Δhfq* is impaired in motility

**Fig. 6:**
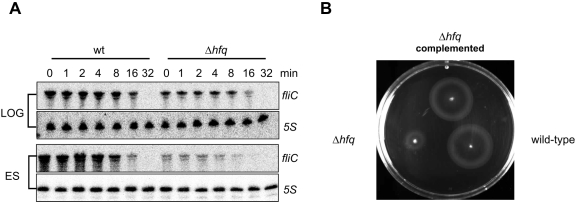
The Δ*hfq* strain is non-motile. A. Northern blot detection of *fliC* mRNA levels in wild-type and Δ*hfq* cells at logarithmic and early stationary phase before and within 32 min after rifampicin treatment. Densitometry of the Northern blot signals showed that the *fliC* mRNA decays with the same half-life in both genetic backgrounds (∼9 min or ∼7 min in logarithmic or early stationary phase cultures respectively). 5S signals are shown as loading control. B. To measure motility, equal numbers of bacteria from each strain were inoculated onto a motility agar-plate. The image was obtained following 4 h of incubation at 37°C.

The strong Hfq dependence for expression of the phase 2 flagellin protein, FliC, suggested that Hfq would be required for *Salmonella* motility. To verify reduced FliC expression, we first analysed *fliC* mRNA levels in wild type and Δ*hfq Salmonella* at different growth phases (Table 3 and Fig. 6A). Interestingly, loss of Hfq caused a mere 1.6-fold reduction of *fliC* mRNA levels in exponential phase, however, a sixfold reduction at early stationary phase (Table 3). We also compared *fliC* mRNA stability in wild-type strain and Δ*hfq* cells, and found it largely unaffected by the *hfq* mutation at either growth phase (Fig. 6A). In contrast to *fliC* mRNA, we failed to detect *fljB* mRNA on any of these Northern blots (data not shown). Taken together, the reduced FliC expression of Δ*hfq* is unlikely to result from phase variation of the invertible flagellar switch (*fljB/fljA* promoter), but rather from reduced *fliC* transcription.

**Table 3:** Quantification of Hfq-dependent gene expression.

	Relative mRNA levels^a^		Relative transcriptional/translational fusion activity^b^
Gene/OD_600_	0.3	2	2
*fliC*	−1.6	−6	ND
*ompC*	1.7	1.6	0.84/1.1
*ompD*	1.7	1.4	0.82/2.5
P_LtetO_-*gfp*	ND	ND	1.0

a Fold change of mRNA levels in *hfq* strain as compared with SL1344 as determined by Northern hybridization.

b Fold change of GFP reporter fusion activity in *hfq* strain as compared with SL1344.

Next, we compared the motility of the wild-type and the Δ*hfq* strains, harbouring either a control or complementation plasmid pStHfq-6H, on motility agar plates. Wild-type cells were motile and formed concentric motility rings around the point of inoculation (Fig. 6B). In contrast, the Δ*hfq* mutant displayed impaired motility, as judged by the much smaller motility ring formed. The strongly reduced motility of Δ*hfq* could also be seen by light microscopy of samples from liquid culture (data not shown). Complementation with plasmid pStHfq-6H fully restored motility. Two control strains, *hfq*-C and *hfq*^HIS^, were found to be as motile as the wild type (data not shown), further supporting that loss of motility was a direct consequence of the lack of Hfq.

### Growth rate-dependent repression of OmpD

**Fig. 7:**
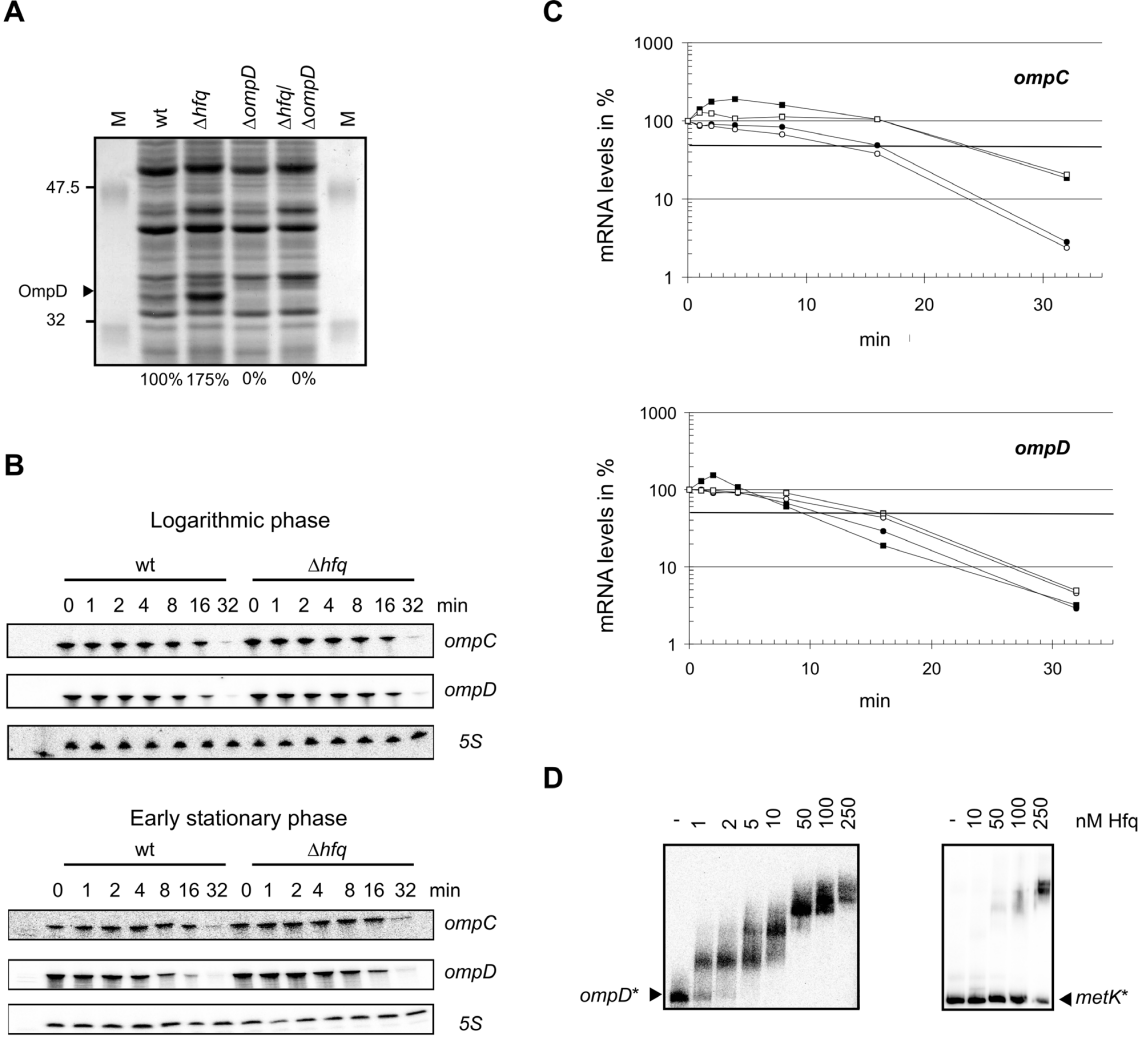
Hfq is essential for growth rate-dependent repression of OmpD. A. SDS-PAGE analysis of total protein prepared from wild-type, Δ*hfq*, Δ*ompD* and Δ*hfq*/Δ*ompD* bacteria grown to early stationary phase. OmpD protein levels as quantified by fluorescent staining (not shown) are given below each lane. B. Northern blot detection of *ompC* and *ompD* mRNA levels of wild-type and Δ*hfq* bacteria grown to either logarithmic or early stationary phase prior to (0 min) and within 32 min of rifampicin treatment. 5S sRNA probing (loading control) is shown below each panel. C. Decay of *ompC* and *ompD* mRNA upon rifampicin treatment as derived from quantification of the Northern blot signals shown in (B). Logarithmic phase, wild-type (filled circles) or Δ*hfq* (open circles); early stationary phase, wild-type (filled squares) or Δ*hfq* (open squares). D. Hfq binds to *ompD* 5′ UTR RNA *in vitro* (gel mobility shift assay). Left panel: 1 nM of ^32^P-labelled *ompD* was incubated with increasing concentrations of Hfq protein (given above the lanes). Following a 15 min incubation at 37°C samples were run on a native 6% gel. Shown is an autoradiograph of the gel. A control gel shift assay with an Hfq-independent RNA derived from the *metK* 5′ UTR is shown in the right panel.

In addition to the positive regulation of secreted effector protein expression, the protein patterns obtained from different growth phases showed that Hfq was also involved in the repression of OmpD synthesis as cells progress into stationary phase (Fig. 3A). To confirm a negative regulatory role for Hfq in OmpD regulation, protein samples of wild-type, Δ*hfq*, Δ*ompD* and Δ*hfq*/Δ*ompD* strains grown to early stationary phase were compared (Fig. 7A). MALDI-TOF analysis of the 40 kDa protein band which showed higher levels of accumulation in the Δ*hfq* strain unequivocally identified it as OmpD, consistent with the complete loss of this protein band in Δ*ompD* and Δ*hfq*/Δ*ompD* cells. Using fluorescent dye staining, we also quantified the relative OmpD accumulation, and found approximately twofold elevated levels of this protein in whole cell lysates (Fig. 7A).

To learn more about the underlying mechanism of Hfq-dependent *ompD* regulation, we first determined the relative changes in *ompC* and *ompD* mRNA abundance at three different points during the growth phase (Table 3). We found that the Δ*hfq* strain exhibited elevated *ompC/D* mRNA levels throughout growth. We also followed the decay of both mRNAs after rifampicin treatment (transcription block, Fig. 7B). Figure 7C shows that absence of Hfq slowed *ompD* mRNA decay twofold (half-lives: ∼9 min versus ∼16 min in wild-type and Δ*hfq* strains), whereas *ompC* decay was not affected.

Next, we constructed transcriptional and translational reporter (GFP) plasmids for both mRNAs. Quantification of GFP reporter activity showed a slightly decreased *ompD* promoter activity (0.82-fold) at early stationary phase, whereas *ompD* translation was upregulated > 2.5-fold (Table 3). As the enhanced activity of the translational *ompD* fusion was consistent with elevated OmpD protein levels (Fig. 7A), we reasoned that Hfq may bind to the 5′ region of the *ompD* mRNA to interfere with its translation. To test this hypothesis, we synthesized a 5′ fragment of the *ompD* mRNA, encompassing its 5′ UTR and 118 nucleotides of the coding region, and performed *in vitro* mobility shift assays with purified Hfq protein. Figure 7D shows that Hfq binds this fragment with high affinity. Up to four different Hfq/*ompD* complexes are observed with increasing Hfq concentration, indicating that there are several Hfq binding sites in the *ompD* 5′ UTR. In contrast, no significant shift was observed with an Hfq-independent RNA (5′ UTR of *metK*) within a 250 nM range of Hfq (Fig. 7D). Taken together, these data suggests a direct role for Hfq in translational repression of the *ompD* mRNA.

## Discussion

The RNA chaperone, Hfq, has recently been recognized as a major post-transcriptional regulator of bacterial gene expression which participates in numerous regulatory pathways (Valentin-Hansen *et al*., 2004). First identified as a host factor for replication of RNA phage Q*β* in *E. coli*, Hfq has been shown to have a broad impact on physiology in several bacteria. The role of Hfq beyond phage replicative functions was first shown with an *E. coli hfq*::Ω mutation, which resulted in pleiotropic phenotypes related mainly to reduced survival of stress conditions (Tsui *et al*., 1994). Later, Hfq was found to be required in *E. coli* and *Salmonella* for efficient translation of *rpoS* mRNA, encoding the general stress sigma factor, σ^S^ (Brown and Elliott, 1996; Muffler *et al*., 1996). As RpoS is required for *Salmonella* proliferation in mice (Fang *et al*., 1992; Nickerson and Curtiss, 1997; Humphreys *et al*., 1999), it has been assumed that Hfq plays an important role in *Salmonella* virulence (e.g. Ding *et al*., 2004). However, the mechanisms by which Hfq affects the pathogenicity of *Salmonella* remained undefined. Previous work in *E. coli* established that Hfq also has regulatory functions independent of its effects on σ^S^ expression (Muffler *et al*., 1997). Likewise, *B. abortus* does not possess an RpoS-like σ factor (Roop *et al*., 2003), yet an *hfq* mutant of *B. abortus* has a pronounced virulence defect (Robertson and Roop, 1999). Similarly, the virulence defect of a *V. cholerae hfq* mutant was not accompanied by reduced σ^S^ levels (Ding *et al*., 2004).

**Fig. 8:**
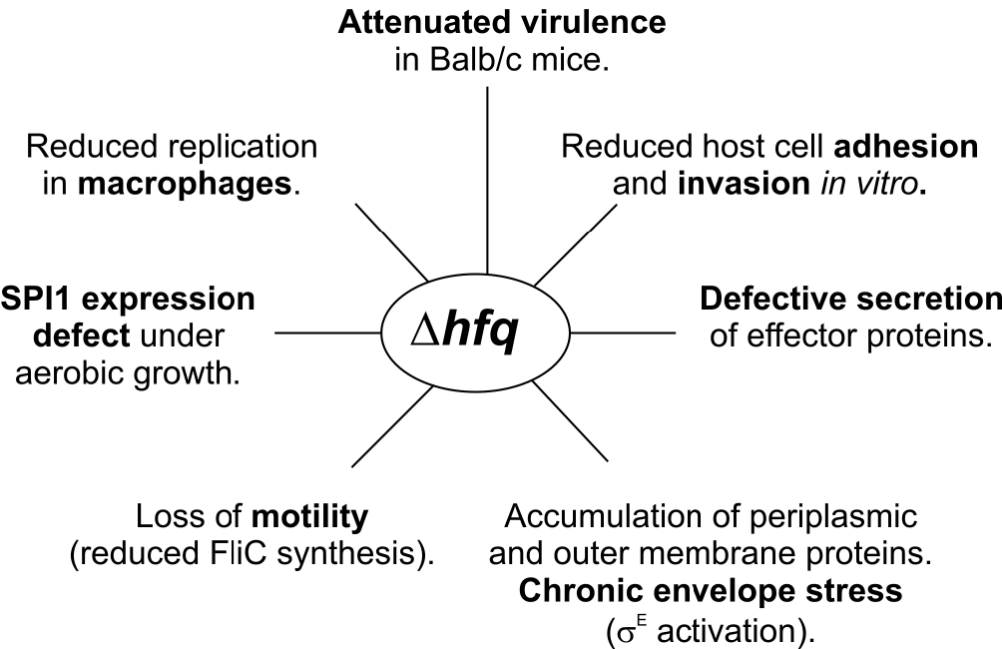
Summary of phenotypes of the *Salmonella hfq* mutation determined in this study.

Peroral infection of the *Salmonella hfq* mutant revealed about the same degree of attenuation (Fig. 2A) as reported for a *Salmonella*Δ*rpoS* mutant, i.e. approximately a three-log difference in cfu recovered from the spleen 3 days post infection using a 10-fold higher infective dose (Nickerson and Curtiss, 1997). Generally, *hfq* mutants of several *Salmonella* strains exhibit four- to sevenfold reduced RpoS levels (Fig. S6; Brown and Elliott, 1996; Bang *et al*., 2005). This is about the degree of RpoS reduction observed in the mouse-avirulent strain, LT2, which has an altered *rpoS* start codon. At first glance, these observations appear to support a model in which reduced σ^S^ production would fully account for the attenuation of Δ*hfq*. However, using a set of newly constructed SL1344 *hfq* mutant and control strains, we defined *hfq* phenotypes that relate to virulence and global gene expression (see Fig. 8 for a summary), and which are largely independent of σ^S^.

The most prominent virulence-associated phenotype we observed is the drastically reduced invasiveness of the Δ*hfq* mutant (Table 1). The ability of *Salmonellae* to invade cultured non-phagocytic cells is dependent on the expression of SPI1-encoded genes (Lee *et al*., 1992), and is strongly dependent on growth rate and media. Two growth conditions showing maximal invasiveness have been defined: growth in LB with aeration to early stationary phase, and growth in low-oxygen, high-salt media (SPI1-inducing). We found that although the *hfq* mutant is defective for invasion under both conditions, the underlying mechanisms are different. When grown to early stationary phase, the Δ*hfq* strain fails to activate the SPI1 transcription factor cascade, characterized by reduced HilA levels and the lack of SPI1 effector protein expression. Our observation that HilA overexpression resulted in the re-appearance of secreted protein expression indicated that the major target of Hfq regulation is HilA activation. This conclusion is also supported by the appearance of normal intracellular levels of SopB and SopE (Fig. 4C), both of which are encoded outside of SPI1 and whose expression requires the concerted function of InvF and SicA. The latter, SPI1-encoded genes are also highly dependent on HilA for expression (Darwin and Miller, 2000, and references therein).

The regulation of *hilA* promoter activity is complex, involving the coactivators HilC, HilD and RtsA, as well as other factors which act upstream of these proteins (Lostroh and Lee, 2001; Ellermeier *et al*., 2005; and references therein)*.* A global transcriptome microarray analysis indicated that Δ*hfq* cells have several-fold reduced levels of *hilC/D* and *rtsA* mRNAs (A. Sittka *et al*., unpubl. results), suggesting that Hfq affects signal transmission further upstream in the SPI1-activating cascade. Strikingly, complementation with the HilA plasmid restored intracellular levels of several effector proteins encoded within SPI1, yet not their secretion. The latter observation may result from a failure to assemble a functional SPI1 TTSS, because only traces of the needle protein, PrgI, were detected in supernatants of HilA-complemented Δ*hfq* cells. The *prgI* gene is encoded within the SPI1 *prgHIJKorgABC* operon (Klein *et al*., 2000), and is directly controlled by HilA. These observations suggest that the role of Hfq as a novel factor of SPI1 gene activation may not be confined to promoting HilA expression. It remains possible that Hfq either is also involved in the mRNA stability of the *prgHIJKorgABC* operon transcript, or affects the translation of the encoded gene products. Further work is required to clarify the effects of Hfq on this subset of HilA-dependent genes.

In contrast to aerobic growth, under SPI1-inducing conditions the Δ*hfq* mutant shows normal SPI1 gene expression, TTSS assembly (as judged by PrgI levels in the supernatant) and effector protein secretion (Fig. 5B). Under these growth conditions, the Δ*hfq* mutant should have been capable of invasion of non-phagocytic cells, yet invasion was strongly reduced compared with the wild-type strain (Table 1). Our results from adhesion and motility assays as well as proteome analysis indicate several other factors may contribute to this impairment. The *hfq* mutant shows a significantly reduced ability to adhere to HeLa cells (Fig. S5), which is likely to affect the rate of invasion. The *hfq* mutant is non-motile (Fig. 6), due most likely to the loss of the flagellar subunit protein, FliC (Figs 3A–C and 6A). However, while flagella-mediated bacterial motility accelerates the invasion of *Salmonella*, motility *per se* is not required for invasion (van Asten *et al*., 2004). Finally, a preliminary proteome analysis (Table 2) showed differential regulation of numerous lipoproteins and OMPs, suggesting that Hfq is also involved in regulation of genes related to the bacterial envelope composition. Importantly, Δ*hfq* cells exhibit strongly elevated levels of HtrA, also known as DegP. HtrA/DegP has recently been shown in *Salmonella* and *E. coli* to be part of the σ^E^ regulon that mediates the response to envelope stress (Rhodius *et al*., 2006; Skovierova *et al*., 2006), and activation of the σ^E^ pathway (by RpoE overexpression) results in a strong induction of *htrA* mRNA (Rhodius *et al*., 2006). Three additional proteins that promote OMP assembly, FkpA, YraP and YaeT, and whose genes are members of the σ^E^ core regulon (Rhodius *et al*., 2006; Skovierova *et al*., 2006), also showed elevated levels in the *hfq* mutant. In addition, two strictly σ^E^-dependent small RNAs, MicA and RybB, showed promoter activation in the *hfq* mutant under the same conditions used in this study (Papenfort *et al*., 2006, and unpublished results). Interestingly, strong induction of the σ^E^ response was also observed in a *V. cholerae hfq* mutant (Ding *et al*., 2004). Based on the activation of multiple σ^E^-dependent genes, the Δ*hfq* strain appears to experience chronic envelope stress which would ultimately change outer membrane properties. In summary, we suggest that the multiple phenotypes of the *hfq* mutant on motility and adherence, and an apparent chronic cell envelope stress in *Salmonella* all contribute to the observed reduced invasiveness of the *hfq* mutant.

A comparison of the *hfq* phenotypes that relate to virulence of *Salmonella* and other previously studied pathogenic bacteria reveals interesting similarities yet also major differences. Hfq mutants of the rather closely related species, *V. cholerae* and *P. aeruginosa*, are severely attenuated for virulence in mice (Sonnleitner *et al*., 2003; Ding *et al*., 2004). In contrast, *hfq* mutants of *L. monocytogenes* and *L. pneumophila* show only mild virulence defects in Balb/c mice and an amoeba infection model respectively (Christiansen *et al*., 2004; McNealy *et al*., 2005). A mouse virulence defect was also described for the *B. abortus hfq* mutant, although Hfq did not appear to affect spleen colonization *per se*, but rather the survival and/or persistence in this organ (Robertson and Roop, 1999). Survival in macrophages was investigated for *L. pneumophila*, *L. monocytogenes* and *B. abortus*, and the effects of the respective *hfq* mutations were comparable to those described here for *Salmonella*, although the *B. abortus hfq* was affected in long-term macrophage survival (Robertson and Roop, 1999; Christiansen *et al*., 2004; McNealy *et al*., 2005). Thus far, *L. monocytogenes* is the only other species for which an *hfq* mutant has been studied with respect to non-phagocytic cell invasion, and unlike *Salmonella*, the *L. monocytogenes hfq* mutant was found to be fully invasive (Christiansen *et al*., 2004). Also in contrast to the *Salmonella hfq* mutant, the assembly of functional pili and secretion of cholera toxin was not affected in the *hfq* mutant of *V. cholerae* (Ding *et al*., 2004). In light of the variability and diversity of Hfq function(s) in virulence among these pathogens, the clear loss of SPI1 expression and the secretion phenotype shown here for *Salmonella* provide an excellent basis to dissect the mechanisms of Hfq functions in a well-characterized model pathogen.

Analyses of protein patterns on one- and two-dimensional gels showed that the expression of a large number of *Salmonella* genes is affected by Hfq. Classification of these genes according to the genome annotation of *Salmonella* LT2 (McClelland *et al*., 2001) shows that the encoded proteins belong to diverse functional categories (Table 2). The increase of GlpK and GlpQ in the *hfq* mutant is currently unexplained, but might indicate changes in glycerophospholipid metabolism (note that the *glpK* and *glpQ* genes are not linked). Other pronounced changes include OMPs such as OmpD, the flagellin FliC, and numerous periplasmic proteins. Given that Hfq has recently been in the spotlight as a small RNA-binding protein (Valentin-Hansen *et al*., 2004), the altered periplasm of Δ*hfq* cells is of particular interest. Specifically, the ∼200 nt GcvB RNA of *E. coli* as well as its *Yersinia pestis* homologue was shown to negatively regulate the periplasmic proteins, OppA, DppA and GltI (Urbanowski *et al*., 2000; McArthur *et al*., 2006), which all accumulate to higher levels in the Δ*hfq* strain (Fig. 3B). The molecular mechanism of GcvB action in these two species remains unknown, but OppA was found to strongly accumulate in an *E. coli*Δ*hfq* mutant (Ziolkowska *et al*., 2006)*.* Moreover, GcvB co-immunoprecipitates with *E. coli* Hfq (Zhang *et al*., 2003), suggesting that this protein mediates GcvB binding to *trans*-encoded target mRNAs. As the *gcvB* gene is conserved and expressed in *Salmonella* (Urbanowski *et al*., 2000; C.M. Sharma and J. Vogel, unpublished), it is tempting to speculate that the high levels of OppA, DppA and GltI observed here results from a loss of GcvB-mediated mRNA repression in the absence of Hfq.

Of the 71 proteins with altered levels in the *hfq* mutant (Table 2), five have no known homologues in *E. coli* (SipA, SipC, STM1254, STM1328 and STM2494). Of the remaining 66, seven overlap with previously published Hfq-associated *E. coli* mRNAs, i.e. CspD, Dps, LppA, LppB, OmpX, RplL and YfiA (Zhang *et al*., 2003). Notably, the majority of these are proteins whose expression was reduced, suggesting Hfq might function to stabilize their mRNAs, either directly or indirectly by promoting efficient translation.

One of the most drastic changes we observed in the absence of Hfq is the increase in OmpD levels (Fig. 7A). OmpD is a *Salmonella*-specific porin, and is the most abundant protein in the outer membrane under standard growth conditions. Together with the other major porins, OmpC and OmpF, it accounts for ∼1–2 × 10^5^ porins per cell (Santiviago *et al*., 2003). Expression of this porin is regulated primarily at the level of transcription, is subject to catabolite repression, and the *ompD* promoter is repressed by low pH. However, post-transcriptional activation of OmpD expression under anaerobiosis has also been reported, and shown to depend on the global transcription regulator, FNR (Santiviago *et al*., 2003), whereas bile appears to repress *ompD* post-transcriptionally (Prouty *et al*., 2004). Despite its abundance, the physiological roles of OmpD remain unclear. Unlike the other two major porins, OmpC and OmpF, OmpD is not regulated by osmolarity (Santiviago *et al*., 2003). The only physiological role of OmpD elucidated thus far is its requirement for the efficient efflux of the toxic compound, methyl viologen (Santiviago *et al*., 2002). In contrast, possible contributions of OmpD to *Salmonella* pathogenicity remain a matter of debate. Two LD_50_ studies of *Salmonella* wild-type and *ompD* mutant strains in mice yielded inconsistent results (Dorman *et al*., 1989; Meyer *et al*., 1998). Other studies postulated a requirement of OmpD for adherence to human macrophages and intestinal epithelial cell lines (Negm and Pistole, 1998; Hara-Kaonga and Pistole, 2004). Intriguingly, the presence of *ompD* correlates with the ability of *Salmonella* serovars to grow in alternative, non-human hosts. Santiviago *et al*. (2003) identified *ompD* in all *Salmonella* serovars that have multiple mammalian hosts, e.g. *S. typhimurium* and *Salmonella enteritidis*, but its absence in *Salmonella typhi*, which is restricted to humans.

In any case, the conservation of *ompD* argues for an important function, and the data obtained here implicate Hfq as a novel factor of *ompD* mRNA regulation at the post-transcriptional level. Hfq binds with high affinity and presumably at multiple sites to the *ompD* 5′ UTR *in vitro*, and its absence stabilizes the *ompD* mRNA *in vivo*. Interestingly, both these observations bear striking similarity to the previously reported Hfq-dependent control of OmpA, the major OMP of *E. coli*, i.e. increased *ompA* mRNA stability in *E. coli hfq* mutants, and Hfq binding of this messenger (Vytvytska *et al*., 1998; Udekwu *et al*., 2005). Importantly, it has recently become clear that one role of Hfq in this regulation may be the promotion of MicA function, an Hfq-dependent sRNA that represses *ompA* mRNA translation in stationary phase (Rasmussen *et al*., 2005; Udekwu *et al*., 2005). There is ample evidence of fine tuning of *E. coli* OMP expression by Hfq-dependent sRNAs. In addition to MicA, six *E. coli* sRNAs, namely MicC, MicF, OmrA/B, RseX and RybB, were shown to mediate repression of single or multiple OMP-encoding mRNAs (reviewed in Guillier *et al*., 2006; Vogel and Papenfort, 2006). Similarly, unpublished results from our laboratory show that *ompD* mRNA is acted upon by the *Salmonella* homologues of the *E. coli* sRNAs, MicC and RybB. In addition, the SPI1-endoded 80 nt InvR RNA negatively regulates *ompD* expression. As all these sRNAs are Hfq-dependent, we hypothesize that the post-transcriptional effect of Hfq on *ompD* expression reported here is mediated by Hfq-dependent regulatory sRNAs.

In summary, this study implicates Hfq as a major post-transcriptional regulator of *Salmonella* gene expression. Unlike other abundant global regulatory proteins, e.g. Fis, IHF, H-NS and HU (Harrison *et al*., 1994; Wilson *et al*., 2001; Schechter *et al*., 2003; Mangan *et al*., 2006), Hfq is primarily known to act at the RNA level. Interestingly, similar to H-NS that recognizes AT-rich sequences in DNA, Hfq binds to AU-rich RNA species. It has recently been proposed that H-NS repression serves to silence newly acquired genomic loci with different GC-content, thus avoiding detrimental consequences from unregulated expression of these genes following their uptake by *Salmonella* (Lucchini *et al*., 2006; Navarre *et al*., 2006). Experiments are currently underway to determine if Hfq plays a similar role by specifically acting on AU-rich mRNAs of newly acquired genes. If so, Hfq may again turn out to be the ‘host factor’ as which it was originally described 40 years ago (Franze de Fernandez *et al*., 1968).

## Experimental procedures

### Oligonucleotides

The complete list of DNA oligonucleotides used for cloning and as probes in hybridization is provided as supplementary material (Table S2).

### Bacterial strains, media and growth conditions

Growth in LB broth or on LB plates at 37°C was used throughout this study unless stated otherwise. SOC medium was used to recover transformants after heat shock or electroporation and prior to plating. Green plates for screening against lysogens in P22 transductions were prepared as described (Sternberg and Maurer, 1991). For SPI1 induction, cultures were inoculated in 5 ml LB containing 0.3 M NaCl in 15 ml Falcon tubes with a tightly closed lid. Cultures were incubated for 12 h at 37°C with shaking. To determine growth rates of strains, the inoculated culture was split in 12 aliquots and each aliquot was opened only once to measure OD_600_. Antibiotics (where appropriate) were applied at the following concentrations: 100 μg ml^−1^ ampicillin, 50 μg ml^−1^ kanamycin, 20 μg ml^−1^ chloramphenicol. For HilA expression from plasmid pCH112, cultures were grown to an OD_600_ of 1 and induced with l-arabinose in a final concentration of 0.05% until cells reached an OD_600_ of 2.

The bacterial strains used in this study are listed in Table 4. Chromosomal mutagenesis of *Salmonella* SL1344 followed the protocol described by Datsenko and Wanner (2000) with few modifications. Strain JVS-00008, which carries plasmid pKD46, was grown in LB at 28°C complemented with ampicillin and 0.2% l-arabinose to an OD_600_ of 0.5. Cells were collected by centrifugation (2 min, 11 000 *g*), washed three times with ice-cold H_2_O, and dissolved in 1/100 of the original culture volume. PCR products of marker genes (50 μl standard reactions) were DpnI-treated for 30 min at 37°C, and purified on Macherey-Nagel spin columns (NucleoSpin Extract II). One-fifth of the 25 μl column eluate (in water) was used for transformation. Forty microlitres of competent cells was mixed with the purified PCR product in a chilled cuvette (0.1 cm electrode gap) and electroporated (18 kV cm^−1^). Subsequently, 1 ml of pre-warmed SOC medium was added, and cells were recovered by incubation for 1 h at 37°C before selection on LB agar plates with the appropriate antibiotics. All mutations were moved to a fresh SL1344 background by phage P22 transduction.

**Table 4:** Strains and plasmids used in this study.

Strain	Relevant markers/genotype	Reference/source
*S. typhimurium*		
SL1344	Str^R^*hisG rpsL xyl*	Hoiseth and Stocker (1981), provided by D. Bumann, MPI-IB Berlin
JVS-00255	SL1344 Δ*hfq::*Cm^R^	This study
JVS-00177	SL1344*hfq*-6HIS-Cm^R^	This study
JVS-00179	SL1344*hfq*-Cm^R^	This study
JVS-00756	SL1344*hilA*-3xFLAG-Km^R^	This study
JVS-00405	SL1344 Δ*spi1* (Km^R^ cassette removed)	S. Pätzold, MPI-IB Berlin (unpublished)
JVS-00748	SL1344 Δ*rpoS*::Km^R^	Kowarz *et al*. (1994)
JVS-00584	SL1344 Δ*hfq* (Cm^R^ cassette removed)	This study
JVS-00735	SL1344 Δ*ompD*::Km^R^	This study
JVS-00822	SL1344 Δ*hfq::*Cm^R^/Δ*ompD*::Km^R^	This study
*E. coli*		
TOP10	*mcr*A Δ(*mrr-hsd*RMS*-mcr*BC) Φ8*0lac*ZΔM15 Δ*lac*X74 *deo*R *rec*A1 *ara*D139 Δ(*ara-leu*)7697*gal*U *gal*K *rps*L *end*A1 *nup*G	Invitrogen
TOP10F′	F′{*lac*I^q^ Tn*10* (Tet^R^)} *mcr*A Δ(*mrr-hsd*RMS*-mcr*BC) Φ8*0lac*ZΔM15 Δ*lac*X74 *deo*R *rec*A1 *ara*D139 Δ(*ara-leu*)7697*gal*U *gal*K *rps*L *end*A1 *nup*G	Invitrogen
ER 2566	F^–^λ^–^*fhuA*2 [*lon*]*ompT lacZ::T7 gene*1 *gal sulA*11 Δ(*mcrC-mrr*) 114*::IS10* *R*(*mcr*-73:*: miniTn*10)2 *R*(*zgb-*210*::Tn*10) (*Tet*^*S*^ ) *endA*1 [*dcm*]	New England Biolabs

To construct the *hfq* deletion strain, the *cat* chloramphenicol-resistance gene was amplified from plasmid pKD3 with oligonucleotides JVO-0252 and JVO-0318. Strains *hfq*-C and *hfq*^HIS^ were constructed in the same way, using primer pairs JVO-0252/JVO-0253 and JVO-0252/JVO-0319 respectively. Mutants were verified by colony PCR using primers JVO-0076/JVO-0077. For removal of the *cat* gene the Δ*hfq* strain was transformed with the FLP helper plasmid pCP20 (for detailed procedure, see Datsenko and Wanner, 2000). The *ompD* deletion strain was constructed by replacing the gene with a kanamycin marker gene amplified from pKD4 with primers JVO-0817/JVO-0818. The deletion mutant was verified using oligonucleotides JVO-0818/0819. Chromosomal FLAG-tagging (3xFLAG) of *hilA* was carried out as described in Uzzau *et al*. (2001), using primers JVO-0837/0838 on template pSUB11. The chromosomal tagging was verified by PCR with oligonucleotides JVO-839/840, and sequencing of the PCR product.

### Plasmids

Plasmids used, and details of their construction are described in Table 5. Maps of selected plasmids are provided in the supplementary material (Fig. S7). *E. coli* TOP10 and TOP10F′ strains were used for cloning. All plasmids were purified using the Machery-Nagel Plasmid QuickPure Kit. To transform *Salmonella* strains, these were rendered competent using the same protocol as described above, except that cells were cultured at 37°C without arabinose.

**Table 5:** Plasmids used in this study.

Name	Fragment	Comment	Origin/marker	Reference
pJV300		ColE1 control plasmid, based on pZE12-luc, P_LlacO_ promoter transcribes a ∼50 nt nonsense transcript (*rrnB* terminator)	ColE1/Amp^R^	This study
pJV859-8	P_LtetO_-*gfp*	GFP control plasmid (constitutive GFP expression)	pSC101*/Cm^R^	Urban and Vogel (2006)
pJV968-1	‘*lacZ*’	ColE control plasmid, carries 1.5 kb internal *lacZ* fragment	ColE1/Amp^R^	Vogel *et al*. (2004)
pVP003	*luc*	Control plasmid; low-copy version of pZE12-luc	pSC101*/Amp^R^	This study
pVP004-1	*Hfq*-6HIS	pStHfq-6H, expresses a HIS-tagged Hfq under control of its own promoter; includes 1014 bp upstream of *hfq* reading frame	pSC101*/Amp^R^	This study
pVP009		Low-copy version of control plasmid pJV300	pSC101*/Amp^R^	This study
pVP012	‘*lacZ*’	Low-copy version of control plasmid pJV968-1	pSC101*/Amp^R^	This study
pVP019	*ompD::gfp*	*ompD* translational GFP fusion plasmid		This study
pVP020	*ompC::gfp*	*ompC* translational GFP fusion plasmid		This study
pAS009	*hfq*	Overexpression plasmid of *Salmonella hfq* (cloned in N-terminal fusion vector pTYB 11)	M13/AmpR	This study
pAS0046	*gfp*	Transcriptional fusions plasmid, based on pJV859-8	pSC101*/Cm^R^	This study
pAS0047-2	P_*hilA*_* -gfp*	*hilA* transcriptional GFP fusion plasmid	pSC101*/Cm^R^	This study
pAS0057-1	P_*ompC*_* -gfp*	*ompC* transcriptional GFP fusion plasmid	pSC101*/Cm^R^	This study
pAS0058-1	P_*ompD*_* -gfp*	*ompD* transcriptional GFP fusion plasmid	pSC101*/Cm^R^	This study
pJU004		GFP control plasmid	pSC101*/Cm^R^	Urban and Vogel (2006)
pBAD/*Myc*-His A		pBAD control plasmid	pBR322/Amp^R^	Invitrogen
pZS*24-MCS1	*luc*	General expression vector	pSC101*/Km^R^	Lutz and Bujard (1997)
pBAD 18-Kn		pBAD control plasmid	pBR322/Km^R^	Guzman *et al*. (1995)
pCH112	P_BAD_-*hilA-Myc-His*	pHilA; *hilA* ORF in pBAD/*Myc*-His	pBR322/Amp^R^	Lostroh *et al*. (2000)
pKD3		Template for mutant construction; carries chloramphenicol cassette	oriRγ/Amp^R^	Datsenko and Wanner (2000)
pKD4		Template for mutant construction; carries kanamycin cassette	oriRγ/Amp^R^	Datsenko and Wanner (2000)
pKD46	P_araB_-*γ-β-exo*	Temperature sensitive *red* recombinase expression plasmid	oriR101/Amp^R^	Datsenko and Wanner (2000)
pCP20		Temperature sensitive FLP recombinase expression plasmid	oriR101/Amp^R^, Cm^R^	Datsenko and Wanner (2000)
pSUB11		Template for mutant construction; 3xFLAG linked to a Km^R^ cassette	R6KoriV, Amp^R^	Uzzau *et al*. (2001)
pZA31-luc	*luc*	General expression plasmid	p15A/Cm^R^	Lutz and Bujard (1997)
pZE12-luc	*luc*	General expression plasmid	ColE1/Amp^R^	Lutz and Bujard (1997)
pTYB-11		Protein overexpression plasmid (IMPACT-CN system)	M13/AmpR	NEB

Control plasmids based on pZE12-luc were constructed as follows: to lower the copy number of plasmid pZE12-luc, the ColE1 origin was swapped to pSC101* by inserting the AvrII-SacI fragment of plasmid pZS*24-MCS1, resulting in pVP003. To obtain plasmid designated pVP012, a low-copy version of control plasmid pJV968-1, the 1.5 kb ‘*lacZ*’ XbaI/XhoI fragment of the latter was introduced into pVP003 by the same enzymes. Note that these plasmids lack the P_LlacO_ promoter region of pZE12-luc, hence the insert is not transcribed.

To express Hfq-6HIS under control of its own promoter, low-copy vector pVP003 was digested with XhoI/XbaI and ligated to a PCR product obtained with the primer pair JVO-0370/0182 (JVO-0370 binds 1014 bp upstream of the *hfq* open reading frame (ORF) in *miaA* while JVO-182 adds a 6HIS-tag sequence followed by a stop codon to the last codon of *hfq*). For clarity, the obtained plasmid, pVP004-1, is designated in figures as pStHfq-6H.

Control plasmid pJV300 was obtained by ligation of a pZE12-luc derived PCR product. The −1 site of promoter P_LlacO_ is fused to the second position of the XbaI site (which is destroyed upon cloning). Transcription from the P_LlacO_ promoter now yields a ∼50 nt nonsense transcript derived from the *rrnB* terminator on pJV300. To obtain a low-copy version of this plasmid, the origin was changed to pSC101* as described above, yielding pVP009.

To clone transcriptional GFP fusions, a PCR fragment was amplified from plasmid pJV859-8 (GFP expression plasmid) using oligonucleotides JVO-0888/pZE-XbaI. JVO-0888 introduces stop codons after a XhoI and NheI site in all three ORFs, a ribosome binding site, a 7 bp spacer, and the sequence of the first six amino acids (aa) of the GFP coding region with a silent mutation at position 6 (T(r)C) to destroy the GFP internal NheI site. Plasmid pJV859-8 was cut XhoI (removing the promoter region, the ribosome binding site and the sequence for the first 142 aa of GFP), gel-purified, and the vector backbone ligated to the PCR fragment digested with the same enzyme. Due to the internal XhoI site in the GFP coding region (cuts in the sequence after aa 142) this leads to a promoterless transcriptional fusion plasmid (used as a negative control plasmid in transcriptional fusion experiments). The resulting plasmid was designated pAS0046. For construction of the *ompC-gfp* transcriptional fusion plasmid pAS0057-1 and the *ompD-gfp* transcriptional fusion plasmid pAS0058-1, pAS0046 was digested with AatII/NheI and ligated to PCR products amplified with primer pairs JVO-0801/0805 and JVO-0806/0807 respectively, cut with the same enzymes.

For translational *ompD::gfp* and *ompC::gfp* fusions, PCR fragments of oligonucleotides JVO-0726/0802 and JVO-0717/0801 respectively, were inserted into plasmid pJV859-8 by AatII*/*NheI cloning, yielding plasmids pVP019 (GFP fusion to 15th aa of OmpD) and pVP020 (GFP fusion to 12th aa of OmpC) respectively.

To overexpress and purify *Salmonella* Hfq protein, the *hfq* coding region was amplified with primer pair JVO-0078/0084. The PCR product was SapI digested and ligated to the N-terminal fusion vector pTYB11 cut with enzymes SapI/SmaI, yielding plasmid pAS009.

### P22 transduction

P22 lysates were prepared from soft agar plate lysates of donor strains using P22 phage HT/105-1 by standard procedures. Transductions were performed as described by Sternberg and Maurer (1991) using P22 phage HT/105-1 and further purified on Green plates. For unknown reasons, we were not able to prepare lysates of the *hfq* deletion mutant, hence Δ*hfq*/P22 lysates were prepared from this strain upon complementation with plasmid pVP004. Transformants were verified by PCR.

### Gentamicin protection (invasion) assays

The invasion assay was performed as described in Isberg and Falkow (1985). HeLa cells (ATCC CCL2) were seeded in RPMI medium (Gibco), supplemented with 10% FCS, 2 mM l-glutamine, 1 mM sodium pyruvate, 50 μM β-mercaptoethanol, and containing 10 μg ml^−1^ penicillin and streptomycin in 12 well plates with a density of 1 × 10^5^ per well the day before or 0.5 × 10^5^ per well 2 days before infection respectively. At the day of infection HeLa cells reached a density of 1–2 × 10^5^. When seeded 2 days before infection medium was changed the day before the assay was performed. One hour prior to infection medium was changed to RPMI containing no antibiotics.

Bacterial cultures were inoculated 1/100 from overnight cultures into fresh medium. For experiments with cultures in early stationary phase cultures were grown in LB (with 50 μg ml^−1^ ampicillin if indicated) at 37°C, 220 rpm, with normal aeration. For experiments with SPI1-induced bacteria, cultures were grown for 12 h in 15 ml Falcon tubes containing 5 ml LB/0.3 M NaCl (with 50 μg ml^−1^ ampicillin if indicated) at 37°C, 220 rpm, under limited oxygen conditions.

HeLa cells were infected with a moi of 10 with 100 μl of bacterial suspension in RPMI medium. The suspension was plated in serial dilutions on LB plates and incubated o/n at 37°C for determination of the input.

Bacterial cells were centrifuged (37°C, 250 *g*, 10 min) onto the HeLa cell monolayer, followed by a 50 min incubation step at 37°C in an atmosphere containing 5% CO_2_. One hour after infection medium was changed to RPMI (containing 50 μg ml^−1^ gentamicin) to kill non-invasive bacterial cells. Incubation was carried on for additional 60 min. After 2 h of infection medium was changed for the 6 h time point to RPMI containing 10 μg ml^−1^ gentamicin and incubation carried on for additional 4 h. For the 2 h time point cells were washed two times in PBS buffer and collected by scraping HeLa cells from the bottom of each well in PBS/0.1% Triton X-100. Dilutions in PBS were plated on LB plates and incubation carried out o/n at 37°C. Six hours after incubation samples for the second time point are treated the same way. Rate of invasion was calculated according to recovered bacterial cells related to the input. Experiments were carried out in duplicates.

### Macrophage survival assay

Infection of macrophage cell lines was performed as described in Thompson *et al*. (2006). The macrophage cell line used was RawB, a derivative of Raw 264.7 (ATCC TIB-71). Macrophages were seeded in 12 well plates 1 day prior to infection at 1 × 10^5^ cells per well. Next day bacteria were harvested for infection at early stationary growth phase (OD_600_∼2–3). Macrophages were infected with a moi of 1. Bacterial cells were centrifuged (37°C, 250 *g*, 10 min) onto the macrophages, followed by a 20 min incubation step at 37°C in an atmosphere containing 5% CO_2_. Thirty minutes in total after infection medium was changed to RPMI (containing 50 μg ml^−1^ gentamicin) to kill non-invasive bacterial cells. Incubation was carried on for additional 30 min. Medium was changed for the 4 and 24 h time points to RPMI containing 10 μg ml^−1^ gentamicin and incubation carried on for additional 3 or 23 h respectively. The number of intracellular bacteria was determined 1, 4 and 24 h after infection and given in per cent related to the input. Experiments were carried out in triplicates and data are representative of two independent experiments.

### HeLa cell adhesion assay

The adhesion assay was performed as described in Hara-Kaonga and Pistole (2004). In brief, bacteria were grown for 12 h under SPI1-inducing conditions. One hundred microlitres of HeLa cells (5 × 10^5^ per ml in RPMI medium) was incubated with 100 μl of bacterial suspension in RPMI medium for 60 min with a moi of 50 at 37°C in 96 well plates. Infections were carried out in triplicates. Non-adherent bacteria were removed by washing cells 4× with 200 μl PBS at 400 *g*. Each sample was resuspended in 50 μl PBS/4% formaldehyde. Each well was sampled three times, and 10 HeLa cells were analysed per sampling. Cells were counted with 1000× magnification using an Eclipse 50i microscope (Nikon).

In a further adhesion assay similar to the macrophage assay by Buchmeier and Heffron (1989), 1 × 10^5^ HeLa cells per well ml^−1^ were infected with a moi of 10 for 30 min with bacteria grown to early stationary phase (bacteria were spun for 10 min on the HeLa cell monolayer followed by 20 min incubation at 37°C). Each well was washed three times with 1 ml PBS and cells were collected by scraping HeLa cells from the bottom of each well in PBS/0.1% Triton X-100. Dilutions in PBS were plated on LB agar and incubation carried out o/n at 37°C. Rate of adhesion and invasion (determined in parallel as above) was calculated according to recovered bacterial cells related to the input. Experiments were carried out in triplicates.

### Animal infections

Bacterial cultures for mice infections were grown in l-broth to early stationary phase (OD_600_ of 2–3), harvested by centrifugation, and diluted to the appropriate cfu ml^−1^ in sterile PBS for infections. For peroral infections, strains were resuspended at 10^9^ cfu ml^−1^, and 0.1 ml of the resuspensions (∼10^8^ bacteria) used to infect groups of five Balb/c mice per strain. The total infective dose was determined in parallel by plating dilutions to agar plates with or without selection, where appropriate. After 72 h, the mice were sacrificed by euthanization in a CO_2_ chamber, and spleens were removed for determination of organ bacterial loads. Isolated spleens were washed once in 70% ethanol, once in PBS and homogenized in 1 ml of PBS. Cell resuspensions were lysed by addition of 1 ml of 0.2% Triton X-100 in deionized, distilled water and incubation at room temperature for 15 min. Dilutions of the cell lysates were plated to agar plates with or without antibiotic selection where appropriate for enumeration of total intracellular bacteria. Intraperitoneal infections were performed by injection of 0.1 ml of a 1:1 mixture of bacterial suspensions of 2 × 10^6^ cfu ml^−1^ of wild-type and mutant strains into the peritoneal space, yielding a final infective dose of approximately 10^5^ cfu ml^−1^ for each strain per animal. Forty-eight hours after the infections, mice were sacrificed and spleens isolated and processed as above. The CI was calculated from the ratios of total input and recovered wild-type and chloramphenicol-resistant Δ*hfq* cfu as previously described (Shea *et al*., 1996).

### Motility assay

Cultures were diluted 1/100 into fresh media and incubated at 37°C/220 rpm to an OD_600_ of 2. One microlitre of culture was inoculated in motility agar plates (LB/0.3% agarose), followed by incubation for 4 h at 37°C.

### Whole cell protein fractions

Culture samples were taken according to 1 OD_600_. Samples were spun 2 min at 16 100 *g* at 4°C. The cell pellet was resuspended in 1× sample loading buffer (1× SLB; Fermentas) to a final concentration of 0.01 OD µl^−1^. Samples were heated 5 min at 95°C. For small and large SDS-PAGE 0.1 OD and 0.2 OD, respectively, were loaded per lane.

### Secreted protein fractions

The protocol for extraction of secreted protein fractions was modified from the protocol described in Kaniga *et al*. (1995). Culture samples were taken either from regular LB cultures at OD 2 or after 12 h of growth or after 12 h of growth in SPI1-induction media, and spun 20 min at 16 100 *g* at 4°C. Proteins from the supernatant were precipitated by adding 25% TCA to a final concentration of 5% followed by 20 min centrifugation at 16 100 *g*, 4°C. The pellet was washed 2× in ice-cold acetone and air dried. The pellet was resuspended in 1× SLB to a final concentration of 1 OD/10 μl. Samples were heated 5 min at 95°C. For small and large SDS-PAGE 1 OD and 2 OD, respectively, were loaded per sample.

### Periplasmic protein fractions

Periplasmic proteins were extracted following the cold osmotic shock procedure described by Neu and Heppel (1965). Overnight cultures were inoculated 1/100 in fresh media and grown to an OD_600_ of 2. Cells were harvested (30 min, 4000 *g*, 4°C) and the pellet was resuspended at room temperature in ‘shock buffer’ (30 mM Tris-HCl, pH 8.0, 20% sucrose). EDTA, pH 8.0 was added at a final concentration of 1 mM. Cells were incubated for 10 min at room temperature with occasional shaking. Cells were collected by centrifugation (30 min, 4000 *g*, 4°C) and the pellet resuspended in 10 ml ice-cold 5 mM MgSO_4_. After incubation for 10 min with occasional shaking in an ice-water bath, the suspension was centrifuged as mentioned above. The supernatant is the cold osmotic shock-fluid.

### Membrane fractions

The total membrane protein fraction was extracted essentially as described (Matsuyama *et al*., 1984). Culture samples were taken at OD_600_ of 2 (4 OD total) and spun 20 min at 16 100 *g* at 4°C. Pellets were washed 1× in 2 ml 10 mM phosphate buffer (pH 7.2). Pellets were resuspended in 0.5 ml of the same buffer. Cells were disrupted by sonication on ice (cycle duty 80%, tip limit 9, four cycles of 30 s with 1 min break on ice). The supernatant was cleared of unbroken cells by centrifugation for 10 min at 1400 *g*, 4°C. Cell envelopes were recovered by centrifugation of the supernatant for 30 min at 16 100 *g*, 4°C. After resuspending the pellet in 2 ml phosphate buffer containing 2% Triton X-100 the samples were incubated for 30 min at 37°C. The insoluble fraction was recovered by 30 min centrifugation at 16 100 *g* at room temperature. After one wash in 2 ml phosphate buffer followed by 5 min centrifugation at 16 100 *g* the pellet was resuspended in 50 μl phosphate buffer (results in approximately 100 μg in 50 μl). Five microlitres per sample was separated on 10% SDS-PAGE.

### Western blot

Commercially available antibodies and antisera used in this study are listed in Table S3. 0.01 or 0.02 OD and 0.1 or 0.2 OD whole cell and secreted protein fractions, respectively, were separated via SDS-PAGE. Proteins were blotted for 60 min at 100 V at 4°C in a cable tank blotter (Peqlab) onto PVDF (Perkin Elmer) membrane in transfer buffer (25 mM Tris base, 190 mM Glycin, 20% Methanol). Blots were rinsed 1× in TBST_20_ buffer (20 mM Tris base, 150 mM NaCl, 0.1% Tween 20). Membranes were blocked for 1 h in 10% dry milk in TBST_20_. Hybridization as follows: appropriate antisera or antibodies (in 3% BSA, TBST_20_; see Table S3 for dilutions) for 1 h at room temperature, 5 × 6 min wash in TBST_20,_α-Rabbit-HRP or α-mouse-HRP (1:5000 in 3% BSA in TBST_20_) for 1 h at room temperature, 6 × 10 min wash in TBST_20_. Blots were developed using Western Lightning (Perkin Elmer) in a Fuji LAS-3000.

### Two-dimensional gel analysis and protein identification

Sample preparation from *Salmonella* cultures at the growth phases given in the respective figure legends, analysis by high-resolution two-dimensional electrophoresis, protein staining, and peptide mass fingerprinting, were performed at the MPI-IB protein analysis core facility (http://info.mpiib-berlin.mpg.de/jungblut/) according to previously published standard protocols (Jungblut and Seifert, 1990; Klose and Kobalz, 1995; Doherty *et al*., 1998; Jungblut *et al*., 2000).

### Protein quantification by fluorescent stain

Cultures of the wild-type, the *hfq* mutant, the *ompD* mutant, and the *hfq/ompD* double mutant strain were grown with aeration at 37°C, 220 rpm to OD 2. Total protein samples corresponding to 0.1 OD culture were separated on SDS-PAGE (15% gel). Gels were stained with Sypro Ruby (Bio-Rad) following the manufacture's protocol. Protein levels were analysed using the fluorescence mode of a phosphorimager (Phosphorimager, FLA-3000 Series, Fuji) using a 473 nm laser and filter O58. Band intensities were quantified with AIDA software (Raytest, Germany).

### Protein overexpression and purification

Overexpression and purification of *Salmonella* Hfq was carried out as published for *E. coli* Hfq (Møller *et al*., 2002) using the IMPACT (Intein Mediated Purification with Affinity Chitin-binding Tag)-CN system (New England Biolabs) according to the manufacture's protocol. Strain ER 2566 carrying plasmid pAS009 was grown to OD of 0.5, and Hfq expression was induced by addition of IPTG (final concentration of 0.5 mM). Following growth for 15 h at 15°C, cells were disrupted using a French press (three passages, 1000 PSI). On-column cleavage of the Hfq moiety was carried out for 24 h at room temperature. The Hfq protein eluate was dialysed against a buffer containing 125 mM NaCl, 12 mM Tris/HCl pH 7.6, 0.5 mM EDTA and concentrated in Vivaspin columns.

### Stability experiments, RNA isolation and Northern detection

Overnight cultures were diluted 1/100 in fresh medium and grown to exponential (OD 0.3) and early stationary phase (OD 2). Rifampicin was added to a final concentration of 500 μg ml^−1^. Incubation was continued at 37°C, 220 rpm, and aliquots (5 ml for OD 0.3; 1.7 ml for stationary phase) were withdrawn prior to or 1, 2, 4, 8, 16 and 32 min after rifampicin addition, mixed with 0.2 vol. of stop solution (5% water-saturated phenol, 95% ethanol), and snap-frozen in liquid nitrogen. After thawing on ice, bacteria were pelleted by centrifugation (2 min, 16 100 *g*, 4°C), and RNA was isolated using the Promega SV total RNA purification kit as described (Kelly *et al*., 2004). The purified RNA was quantified on a Nanodrop machine (NanoDrop Technologies).

RNA samples (∼5 μg) were denatured for 5 min at 95°C in loading buffer containing 95% formamide, separated on 8.3 M urea – 5% polyacrylamide gels (PAGE), and transferred to Hybond-XL membranes (GE Healthcare) by electro-blotting (1 h, 50 V, 4°C) in a tank blotter (Peqlab). Membranes were hybridized at 42°C with gene-specific [^32^P] end-labelled oligodeoxyribonucleotides, random-labelled PCR fragments, or at 70°C with riboprobes, in Rapid-hyb Buffer (GE Healthcare).

*ompC* transcripts were detected with a random-labelled ([^32^P] dCTP; Rediprime II labelling kit, GE Healthcare) PCR fragment generated with primer pair JVO-0717/0719. To detect the *ompD* and *hilA* mRNAs, PCR fragments generated with primer pairs JVO-0751/0934 and JVO-1298/1299, respectively, were *in vitro* transcribed from the T7 promoter (added by primers JVO-0934 and JVO-1299) in the presence of [^32^P]-α-UTP using Ambion's T7 polymerase Maxiscript kit. Riboprobes were purified over a G50 column. *fliC* and *fljB* transcripts were probed using end-labelled oligodeoxyribonucleotides JVO-1592 and JVO-1595. For normalization of RNA amounts 5S signals were detected using end-labelled oligodeoxyribonucleotide JVO-0322. Following hybridization for 2 h, membranes hybridized with riboprobes were washed at 65°C in three subsequent 15 min steps in SSC (2×, 1× or 0.5×)/0.1% SDS solutions, after rinsing the membrane first in 2× SSC/0.1% SDS. Membranes hybridized with PCR fragments were rinsed in 2× SSC/0.1% SDS, followed by 15 min washes in 2× (65°C), 1× and 0.5× (42°C) SSC/0.1% SDS. For end-labelled oligodeoxyribonucleotides hybridization membranes were rinsed in 5× SSC followed by three wash steps at 42°C in SSC (5×, 1× and 0.5× respectively). Signals were visualized on a phosphorimager (Phosphorimager, FLA-3000 Series, Fuji), and band intensities quantified with AIDA software (Raytest, Germany).

### Gel mobility shift assay

*The ompD* DNA template for *in vitro* transcription with T7 RNA polymerase was generated with the primers JVO-1186/-1058. It starts with a T7 promoter fused to the +1 transcriptional start site of OmpD (mapped with 5′RACE; V. Pfeiffer *et al*., in preparation) at position −69 relative to the *ompD* AUG start codon, and ends with the 39th codon of the *ompD* coding sequence. *In vitro* transcription was performed using the Megascript kit (Ambion, #1333), followed by DNase I digestion (1 unit, 15 min, 37°C). Following extraction with phenol : chloroform : isopropanol (25:24:1 v/v), the RNA was precipitated overnight at −20°C with 1 vol. of isopropanol. RNA integrity was checked on a denaturing polyacrylamide gel. 20 pmol RNA was dephosphorylated with 10 units of calf intestine alkaline phosphatase (New England Biolabs) in a 20 μl reaction at 37°C for 1 h. Following phenol extraction, the RNA was precipitated overnight with ethanol/sodium acetate and 20 μg glycogen. The dephosphorylated RNA was 5′ end-labelled with ^32^P-γATP (20 μCi), using 1 unit of polynucleotide kinase (New England Biolabs) for 30 min at 37°C in a 20 μl reaction. Unincorporated nucleotides were removed using Microspin^TM^ G-50 Colums (GE Healthcare), followed by purification of the labelled RNA on a denaturing polyacrylamide gel (6%/7 M urea). Upon visualization of the labelled RNA by exposure on a phosphorimager, the RNA was cut from the gel and eluted with RNA elution buffer (0.1 M sodium acetate, 0.1% SDS, 10 mM EDTA) at 4°C overnight, followed by phenol extraction and precipitation as before.

Binding assays were performed in 1× structure buffer (100 mM Tris pH 7, 1 M KCl, 100 mM MgCl_2_, provided along with RNase T1 from Ambion #2283) as follows: 5′-labelled RNA (0.01 pmol of *ompD* mRNA; final concentration in binding reaction: ∼1 nM) and 1 μg of yeast RNA (final concentration: 4.3 μM) were incubated with increasing concentrations of Hfq in 10 μl reactions at 37°C for 15 min. The Hfq dilutions (1, 2, 3.9, 7.8, 15.6, 31.3, 62.5, 125, 250, 500 or 1000 nM; calculated for the Hfq hexamer) were prepared in 1× dilution buffer (1× structure buffer with 1% glycerol, 0.1% Triton X-100). Prior to gel run, the binding reactions were mixed with 3 μl of loading buffer (50% glycerol, 0.5× TBE, 0.2% bromphenolblue), and electrophoresed on native 6% polyacrylamide gels in 0.5× TBE buffer at 300 V at 4°C for 3 h. Gels were dried, and analysed using a phosphorimager (see above).

To synthesize the Hfq-independent *metK* control RNA, a DNA template for T7 RNA polymerase *in vitro* transcription was amplified with primers JVO-1701/1702. The resulting RNA spans the entire 5′ UTR (129 nt) according to the +1 transcriptional start site mapped in Wei and Newman (2002) and 80 bp of the *metK* coding region. *In vitro* transcription and the labelling reaction were performed as described for *ompD* RNA.

### Fluorescence measurements

Strains carrying the GFP fusion plasmids were inoculated from single colonies in 20 ml LB medium supplemented with 20 μg ml^−1^ chloramphenicol and incubated with aeration at 37°C/220 rpm. At the indicated cell density, 3 × 100 μl culture were transferred to a 96 well plate, and fluorescence was measured at 37°C using a VICTOR^TM^_3_ machine (1420 Multilable Counter, Perkin Elmer). All experiments were done in triplicates. Plasmid pJV859-8, which expresses GFP from a constitutive P_LtetO_ promoter, served as a control. In transcriptional fusion studies, strains carrying plasmid pAS0046 served as background control, while plasmid pJU004 was used in translational fusion studies. A detailed protocol of fluorescence measurement will be described elsewhere (Urban and Vogel, 2006).

## Supplementary material

The following supplementary material is available for this article online:

Table S1 Summary of Hfq-dependent changes of protein expression.

Table S2 Oligonucleotides used in this study.

Table S3 Commercially available antibodies and antisera used in this study.

Fig. S1 Growth characteristics of Salmonella strains under SPI1-inducing conditions.

Fig. S2 The Dhfq mutant is defective for invasion and intracellular replication.

Fig. S3 The Dhfq strain shows an invasion and intracellular growth defect in intestinal epithelial cells and J774A murine macrophage.

Fig. S4 The hfq mutation leads to various differences in protein levels.

Fig. S5 The hfq mutant shows reduced adhesion.

Fig. S6 RpoS expression is Hfq-dependent in SL1344.

Fig. S7 Physical maps of plasmids.

This material is available as part of the online article from http://www.blackwell-synergy.com
