# Supplementary material for: The RNA chaperone Hfq is essential for the virulence of Salmonella typhimurium
Source: Mol Microbiol. 2007 Jan;63(1):193–217. doi: 10.1111/j.1365-2958.2006.05489.x (PMC1810395; doi:10.1111/j.1365-2958.2006.05489.x)
Supplement: Table S1 — Summary of Hfq-dependent changes of protein expression. [file MMI5489TablesS1-S3.doc]

**Supplementary material**

**The RNA chaperone Hfq is essential for the virulence of**

***Salmonella typhimurium***

**Alexandra Sittka**^1^**, Verena Pfeiffer**^1^**, Karsten Tedin**^2^ **and Jörg Vogel**^1^

^1^Max Planck Institute for Infection Biology, RNA Biology Group, Berlin, Germany;

2 Institut für Mikrobiologie und Tierseuchen, Freie Universität Berlin, Berlin, Germany

**Table S1: Summary of Hfq-dependent changes of protein expression.**

| **Name^a^** | **Reg^b^** | **Loc^c^** | **Function^d^** | **Functional class^e^** | **mRNA^f^** | **location^g^** | **spot^h^** | **MW^i^** |
| --- | --- | --- | --- | --- | --- | --- | --- | --- |
| CarA | **-** | CP | carbamoyl-phosphate synthetase, glutamine-hydrolysing small subunit | AMINO ACID TRANSPORT & METABOLISM / NUCLEOTIDE TRANSPORT & METABOLISM |  | 75880..77028 | **44 c+d** | 41625 |
| SurA | **+** | CP | peptidyl-prolyl cis-trans isomerase, survival protein | POSTTRANSLATIONAL MODIFICATION, PROTEIN TURNOVER, CHAPERONES |  | (104470..105756) | **50**  **and Fig4B** | 47221 |
| HtrA | **+** | PP | periplasmic serine protease Do, heat shock protein | POSTTRANSLATIONAL MODIFICATION, PROTEIN TURNOVER, CHAPERONES |  | 244492..245919 | **51**  **and Fig. 4B** | 49284 |
| PyrH | **-** | CP | uridine 5'-monophosphate kinase | NUCLEOTIDE TRANSPORT & METABOLISM |  | 256276..257001 | **22** | 25939 |
| Upp | **-** | CP | undecaprenyl pyrophosphate synthetase (di-trans,poly-cis-decaprenylcistransferase); uracil phosphoribosyltransferase | NUCLEOTIDE TRANSPORT & METABOLISM |  | 259355..260113 | **21** | 22519 |
| YaeT | **+** | (OM) | putative outer membrane antigen | CELL WALL/MEMBRANE BIOGENESIS |  | 262379..264793 | **39+58** | 89471 |
| GltI | **+** | PP | ABC transporter periplasmic binding protein; ABC superfamily, glutamate/aspartate transporter | AMINO ACID TRANSPORT & METABOLISM / SIGNAL TRANSDUCTION MECHANISMS |  | (728909..729835) | **26+27**  **and Fig. 4B** | 34111 |
| SucD | **-** | CP | succinyl-CoA synthetase, alpha subunit | ENERGY PRODUCTION & CONVERSION |  | 807077..807946 | **31** | 29757 |
| Pal | **+** | PP | tol protein required for outer membrane integrity, uptake of group A colicins, & translocation of phage DNA to cytoplasm; peptidoglycan-associated lipoprotein precursor | CELL WALL/MEMBRANE BIOGENESIS |  | 817291..817815 | **10** | 18853 |
| YbgF | **-** | (PP) | putative periplasmic protein | FUNCTION UNKNOWN |  | 817825..818613 | **29** | 28230 |
| Dps | **-** | CP | stress response DNA-binding protein; starvation induced resistance to H2O2; DNA protection during starvation protein | INORGANIC ION TRANSPORT & METABOLISM | + | (897487..897990) | **14** | 18706 |
| CspD | **+** | CP | cold shock-like protein CspD; similar to CspA but not cold shock induced | no COG | + | (1021802..1022023) | **2** | 7880 |
| TrxB | **-** | CP | thioredoxin reductase; thioredoxin reductase | POSTTRANSLATIONAL MODIFICATION, PROTEIN TURNOVER, CHAPERONES |  | (1035228..1036196) | **33** | 34662 |
| FabF | **-** | CP | 3-oxoacyl-[acyl-carrier-protein] synthase II | LIPID TRANSPORT & METABOLISM / SECONDARY METABOLITES BIOSYNTHESIS, TRANSPORT & CATABOLISM |  | 1280435..1281676 | **48** | 42926 |
| IcdA | **+** | CP | isocitrate dehydrogenase in e14 prophage, specific for NADP+ | ENERGY PRODUCTION & CONVERSION |  | 1324385..1325635 | **46** | 45759 |
| PagC | **+** | OM | PhoP regulated: reduced macrophage survival; virulence membrane protein PagC precursor | CELL WALL/MEMBRANE BIOGENESIS | not in  *E. coli* | 1332251..1332808 | **12** | 20171 |
| STM1254 | **-** | (OM) | putative outer membrane lipoprotein | no COG | not in  *E. coli* | (1338127..1338441) | **55** | 10626 |
| STM1328 | **-** | (OM) | putative outer membrane protein | FUNCTION UNKNOWN | not in  *E. coli* | (1407118..1408077) | **29** | 33818 |
| AroD | **-** | CP | 3-dehydroquinate dehydratase | no COG |  | (1439617..1440375) | **24** | 27308 |
| LppB | **-** | OM | putative methyl-accepting chemotaxis protein; major outer membrane lipoprotein | no COG | + | (1459057..1459296) | **5** | 8472 |
| LppA | **-** | OM | murein lipoprotein, links outer & inner membranes; major outer membrane lipoprotein | no COG | + | (1459379..1459615) | **1** | 8386 |
| YnaF | **-** | CP | putative universal stress protein | SIGNAL TRANSDUCTION MECHANISMS |  | 1746131..1746565 | **7** | 15704 |
| Tpx | **+** | CP | thiol peroxidase | POSTTRANSLATIONAL MODIFICATION, PROTEIN TURNOVER, CHAPERONES |  | 1775584..1776090 | **25** | 18014 |
| TrpB | **-** | CP | Tryptophan synthase beta chain | AMINO ACID TRANSPORT & METABOLISM |  | 1823079..1824272 | **44 a** |  |
| OppA | **+** | PP | ABC superfamily, oligopeptide transport protein with chaperone properties | AMINO ACID TRANSPORT & METABOLISM |  | (1839608..1841356) | **54**  **and Fig. 4B** | 65526 |
| KdsA | **-** | CP | 3-deoxy-D-manno-octulosonic acid 8-P synthetase | CELL WALL/MEMBRANE BIOGENESIS |  | (1870407..1871261) | **30** | 30776 |
| PrsA | **-** | CP | phosphoribosylpyrophosphate synthetase | NUCLEOTIDE TRANSPORT & METABOLISM / AMINO ACID TRANSPORT & METABOLISM |  | 1877761..1878708 | **33** | 34195 |
| FliC | **-** | OM / SUP | flagellin, filament structural protein | CELL MOTILITY |  | (2047658..2049145) | **45** | 51581 |
| Gnd | **-** | CP | gluconate-6-phosphate dehydrogenase, decarboxylating | CARBOHYDRATE TRANSPORT & METABOLISM |  | (2159361..2160767) | **47** | 51363 |
| GlpQ | **+** | PP | glycerophosphodiester phosphodiesterase, periplasmic | ENERGY PRODUCTION & CONVERSION |  | (2387530..2388600) | **37+38**  **and Fig. 4B** | 40399 |
| AckA | **-** | CP | acetate kinase A (propionate kinase 2) | ENERGY PRODUCTION & CONVERSION |  | 2447939..2449141 | **44 c** | 43230 |
| HisJ | **-** | PP | ABC superfamily, histidine-binding periplasmic protein | AMINO ACID TRANSPORT & METABOLISM / SIGNAL TRANSDUCTION MECHANISMS |  | (2464491..2465273) | **24** | 28362 |
| CysP | **+** | PP | ABC superfamily, thiosulfate transport protein | INORGANIC ION TRANSPORT & METABOLISM |  | (2555425..2556441) | **32** | 37559 |
| MaeB | **+** | CP | paral putative transferase; phosphate acetyltransferase | ENERGY PRODUCTION & CONVERSION |  | (2579979..2582258) | **56** | 82270 |
| NlpB | **+** | OM | lipoprotein-34 | CELL WALL/MEMBRANE BIOGENESIS |  | (2602992..2604026) | **36** | 36915 |
| STM2494 | **+** | (IM) | putative inner membrane or exported | GENERAL FUNCTION PREDICTION ONLY | not in  *E. coli* | 2607971..2609434 | **52** | 53707 |
| NifU | **-** | CP | NifU homolog involved in Fe-S cluster formation | ENERGY PRODUCTION & CONVERSION |  | (2681123..2681509) | **6** | 13812 |
| YfiA | **-** | CP | ribosome associated factor, stabilizes ribosomes against dissociation; putative sigma(54) modulation protein | TRANSLATION | + | 2807668..2808006 | **4** | 12645 |
| LuxS | **-** | CP | quorum sensing protein, produces autoinducer - acyl-homoserine lactone-signaling molecules | no COG |  | (2966270..2966785) | **19** | 19296 |
| SipA | **-** | SUP | cell invasion protein | no COG | not in  *E. coli* | (3024679..3026736) | **57** | 73897 |
| SipC | **-** | SUP | cell invasion protein | no COG | not in  *E. coli* | (3027857..3029086) | **43** | 42957 |
| GudD | **-** | CP | D-glucarate dehydratase | CELL WALL/MEMBRANE BIOGENESIS / GENERAL FUNCTION PREDICTION ONLY |  | (3110539..3111879) | **49** | 49143 |
| Ptr | **+** | PP | protease III | POSTTRANSLATIONAL MODIFICATION, PROTEIN TURNOVER, CHAPERONES |  | (3149646..3152534) | **59** | 107419 |
| OmpX | **-/+** | OM | ail & ompX Homolog; outer membrane protein x precursor | no COG | + | (3193102..3193638) | **8/9** | 18483 |
| YraP | **+** | (PP) | paral putative periplasmic protein; possible lipoprotein | GENERAL FUNCTION PREDICTION ONLY |  | 3436344..3436919 | **11** | 20085 |
| RbfA | **-** | CP | ribosome-binding factor, role in processing of 10S rRNA | TRANSLATION |  | (3452988..3453389) | **7** | 15156 |
| GreA | **+** | CP | transcription elongation factor, cleaves 3' nucleotide of paused mRNA | TRANSCRIPTION |  | (3467930..3468406) | **13** | 17645 |
| Mdh | **-/+** | CP | malate dehydrogenase | ENERGY PRODUCTION & CONVERSION |  | (3526676..3527614) | **35/34** | 32455 |
| AccB | **+** | CP | acetylCoA carboxylase, BCCP subunit, biotin carboxyl carrier protein | LIPID TRANSPORT & METABOLISM |  | 3550095..3550565 | **16+20** | 16676 |
| FkpA | **+** | CP | FKBP-type peptidyl-prolyl cis-trans isomerase (rotamase) | POSTTRANSLATIONAL MODIFICATION, PROTEIN TURNOVER, CHAPERONES |  | (3604871..3605689) | **28** | 28927 |
| DppA | **+** | PP | ABC superfamily, dipeptide transport protein | AMINO ACID TRANSPORT & METABOLISM |  | (3814422..3816029) | **53 a+b**  **and Fig. 4B** | 60181 |
| YiaD | **+** | (OM) | putative outer membrane lipoprotein | CELL WALL/MEMBRANE BIOGENESIS |  | 3832806..3833468 | **17** | 22291 |
| Kbl | **-** | CP | 2-amino-3-ketobutyrate CoA ligase (glycine acetyltransferase) | COENZYME TRANSPORT & METABOLISM |  | (3903917..3905113) | **44 a+b** | 43004 |
| PstS | **+** | PP | ABC superfamily, high-affinity phosphate transporter | INORGANIC ION TRANSPORT & METABOLISM |  | (4063585..4064625) | **40** | 36794 |
| RbsB | **+** | PP | ABC superfamily, D-ribose transport protein; D-ribose-binding periplasmic protein | CARBOHYDRATE TRANSPORT & METABOLISM |  | 4094587..4095477 | **23** | 30943 |
| FadA | **-** | CP | 3-ketoacyl-CoA thiolase; (thiolase I, acetyl-CoA transferase), small (beta) subunit of the fatty acid-oxidizing multienzyme complex | LIPID TRANSPORT & METABOLISM |  | (4188137..4189300) | **42** | 40978 |
| RplL | **-** | CP | 50S ribosomal subunit protein L7/L12 | TRANSLATION | + | 4365225..4365590 | **3** | 10805 |
| MalE | **-** | PP | ABC superfamily maltose transport protein, substrate recognition for transport & chemotaxis | CARBOHYDRATE TRANSPORT & METABOLISM |  | (4449329..4450528) | **41** | 43456 |
| AphA | **+** | PP | non-specific acid phosphatase/phosphotransferase, class B | GENERAL FUNCTION PREDICTION ONLY |  | 4470683..4471396 | **60** | 26298 |
| OsmY | **-** | PP | hyperosmotically inducible periplasmic protein, RpoS-dependent stationary phase gene | GENERAL FUNCTION PREDICTION ONLY |  | 4815879..4816496 | **18** | 21436 |
| Tsf | **+** | CP | protein chain elongation factor EF-Ts | no COG |  | 255280..256131 | **Fig.4B** | 30339 |
| CyoA | **+** | M | cytochrome o ubiquinol oxidase subunit II | ENERGY PRODUCTION & CONVERSION |  | (497191..498147) | **Fig.4B** | 35270 |
| YbfM | **+** | (OM) | putative outer membrane protein | no COG |  | 749534..750940 | **Fig.4B** | 52631 |
| GlnH | **+** | PP | ABC superfamily (bind_prot), glutamine high-affinity transporter | AMINO ACID TRANSPORT & METABOLISM / SIGNAL TRANSDUCTION MECHANISMS |  | (896264..897010) | **Fig.4B** | 27245 |
| OmpF | **+** | OM | outer membrane protein 1a (ia;b;f), porin | CELL WALL/MEMBRANE BIOGENESIS |  | (1089781..1090872) | **Fig.4B** | 40266 |
| MglB | **+** | PP | ABC superfamily (peri_perm), galactose transport protein | CARBOHYDRATE TRANSPORT & METABOLISM |  | (2286618..2287616) | **Fig.4B** | 35791 |
| STM2786 | **+** | PP | tricarboxylic transport | FUNCTION UNKNOWN | not in  E. coli | 2934590..2935567 | **Fig.4B** | 35457 |
| RpsD | **-** | CP | 30S ribosomal subunit protein S4 | TRANSLATION |  | (3584316..3584936) | **Fig.4B** | 23471 |
| RplC | **-** | CP | 50S ribosomal subunit protein L3 | TRANSLATION | + | (3595557..3596186) | **Fig.4B** | 22234 |
| GlpK | **+** | CP | glycerol kinase | ENERGY PRODUCTION & CONVERSION |  | (4294342..4295850) | **Fig.4B** | 56016 |
| TufB | **-** | CP | protein chain elongation factor EF-Tu (duplicate of tufA) | TRANSLATION |  | 4360603..4361787 | **Fig.4B** | 47221 |

a Candidate protein name according to coliBASE (<http://colibase.bham.ac.uk/index.cgi?help=searchbox&frame=genome>; (Chaudhu*ri et a*l., 2004)).

b Up- or down-regulation in *hfq* strain as compared to SL1344.

c Predicted cellular protein localization, CP (cytoplasm), PP (periplasm), OM (outer membrane), IM (inner membrane), SUP (secreted into supernatant).

d Protein function according to KEGG (<http://www.genome.jp/kegg/>; (Go*to et a*l., 1997)).

e Functional classification according to (McClella*nd et a*l., 2001)

f + indicates Hfq co-immunoprecipitation of the mRNA in E. coli (Zha*ng et a*l., 2003)

g Genomic localization according to (McClella*nd et a*l., 2001). Numbers in parentheses indicate counter-clockwise orientation of the gene.

h Spot number according to Figure S2 or the bands labeled in Fig. 4.

i Protein molecular weight according to (Chaudhu*ri et a*l., 2004).

**Table S2: Oligonucleotides used in this study.**

| **Name** | **Sequence^a^** |
| --- | --- |
| JVO-0076 | GAAGTATTACAGGTTGTTGGTG |
| JVO-0077 | GCATCATAACGGTCAAACA |
| JVO-0078 | GGTGGTTGCTCTTCCAACATGGCTAAGGGGCAATCTTT |
| JVO-0084 | TTATTCAGTCTCTTCGCTGTCCT |
| JVO-0182 | GTTTTTTCTAGATTAATGATGATGATGATGATGTTCAGTCTCTTCGCTGTCC |
| JVO-0252 | GCGATTATCCGACGCCCCCGACATGGATAAACAGCGCGTGAAGTGTAGGCTGGAGCTGCTTC |
| JVO-0253 | ACGCGCAGGGGTCTACTGCGCAACAGGACAGCGAAGAGACTGAATAAGGTCCATATGAATATCCTCCTTAG |
| JVO-0318 | CAGAATCGAAAGGTTCAAAGTACAAATAAGCATATAAGGAAAAGAGGTCCATATGAATATCCTCCTTAG |
| JVO-0319 | ACGCGCAGGGGTCTACTGCGCAACAGGACAGCGAAGAGACTGAACATCATCATCATCATCATTAAGGTCCATATGAATATCCTCCTTAG |
| JVO-0322 | CTACGGCGTTTCACTTCTGAGTTC |
| JVO-0370 | GTTTTTCTCGAGCCTGCCTAAGGC |
| JVO-0397 | CGGTAGAGTAACTATTGAGCAGAT |
| JVO-0398 | GTTTTTTTTTTAATACGACTCACTATAGGGAGGCCTAACCAGTCGTAGC |
| JVO-0717 | GTTTTTGCTAGCTGGTACCAGGAGGG |
| JVO-0719 | GTTTTATGCATGCCGACTGGTTAATGAG |
| JVO-0726 | GTTTTTGCTAGCCAACAGGGAAGTCAC |
| JVO-0751 | GTTTTATGCATAGTCTGCCATTGACAAAC |
| JVO-0801 | GTTTTGACGTCTATTTGTGCTTATTTTTACTTG |
| JVO-0802 | GTTTTGACGTCAAATCAATATTGAAACGG |
| JVO-0805 | GTTTTGCTAGCATGCCTTTATTGCTTTTTTATG |
| JVO-0806 | GTTTTGACGTCTCGACCCGCTGTACCT |
| JVO-0807 | GTTTTGCTAGCGCGTGTTTCCTCAACCA |
| JVO-0811 | GGAGATCTCGATCACACAAATTAAAATAATTTGTAATCGTGTAGGCTGGAGCTGCTTC |
| JVO-0812 | CCAGCCCTGAAAGGACTGGCTTTGTATTCAGACTACAACAAAAGGTCCATATGAATATCCTCCTTAG |
| JVO-0817 | GTTTTTCTCGAGCCAATAGTCCCCTCCGA |
| JVO-0818 | GTTTTTTCTAGACTGCACGGCATACTCCT |
| JVO-0837 | CAAAAGATGGAAACAGGATCCCCGCTTGATTAAATTACGGGACTACAAAGACCATGACGG |
| JVO-0838 | ATGATAAAAAAATAATGCATATCTCCTCTCTCAGATTTTACCATATGAATATCCTCCTTAG |
| JVO-0839 | CCACGGCGAAGCTATT |
| JVO-0840 | GCCCATGCCGTATTTAT |
| JVO-0888 | GTTTTCTCGAGGCTAGCTAAGTAGTACTTAGATTTAAGAAGGAGATATACATATGGCCAGCAAAGGAGAA |
| JVO-0889 | GTTTTTGACGTCAGCGTAAGAATTCGTCC |
| JVO-0890 | GTTTTTGCTAGCTATCTTACTGCATTTTTTT |
| JVO-0934 | GTTTTTTTTTTAATACGACTCACTATAGGGAGGTTAACTGATCGTTGATCTG |
| JVO-1058 | CGTGAACTTTACCGTACA |
| JVO-1186 | TTTTCTCGAGTTAATACGACTCACTATAGGCCATTGACAAACG |
| JVO-1298 | CGAGCCCGTAGAATATGA |
| JVO-1299 | GTTTTTTTTTTAATACGACTCACTATAGGGAGGCTTCGAGCAGGATG |
| JVO-1592 | GCGTATCCAGACCCAGGGTCTGAGAG |
| JVO-1595 | GCGCCAGCCGCAAGGGT |
| JVO-1701 | GTTTTTTTTAATACGACTCACTATAGGATCCATCCATACTGATTAACACT |
| JVO-1702 | GCAGGATAGCGTCCAAC |
| pZE-Xba | TCGTTTTATTTGATGCCTCTAGA |

a Oligonucleotides used for cloning, and Northern hybridization. Sequences are given in 5'->3' direction.

**Table S3: Commercially available antibodies and anti sera used in this study**

| **Epitope** | **Antibody/anti sera** | **Working dilution** | **Source** | **Provided by** |
| --- | --- | --- | --- | --- |
| Myc | α-Myc antibody | 1:1000 | mouse | Santa Cruz Biotechnology, Inc. |
| 3xFLAG | α-FLAG antibody | 1:1000 | mouse | Sigma |
| IgG | α−rabbit IgG antibody HRP-linked | 1:5000 | donkey | Amersham |
| IgG | α−mouse IgG antibody HRP-linked | 1:5000 | sheep | Amersham |
| SipC | α-SipC antiserum | 1:3000 | rabbit | MPI-IB Berlin, Michael Kolbe |
| SipD | α-SipD antiserum | 1:3000 | rabbit | MPI-IB Berlin, Michael Kolbe |
| SopB | α-SopB antiserum | 1:3000 | rabbit | MPI-IB Berlin, Michael Kolbe |
| SopE | α-SopE antiserum | 1:15000 | rabbit | ETH Zurich, Wolf-Dietrich Hardt |
| PrgI | α-PrgI antiserum | 1:3000 | rabbit | MPI-IB Berlin, Michael Kolbe |
| RpoS | α-RpoS antiserum | 1:5000 | rabbit | FU Berlin, Regine Hengge |

**Figure legends**

**Figure S1. Growth characteristics of *Salmonella* strains under SPI1-inducing conditions.**

(A) OD_600_ values of triplicate cultures in LB medium containing 0.3M sodium chloride and grown under oxygen limitation were determined in 60 minute intervals (open squares: wild-type, filled triangles: *hfq*^HIS^, open diamonds: *hfq*-C, stars: Δ*hfq*). (B) Complementation of the slight growth defect of the Δ*hfq* strain by plasmid pStHfq-6H when grown under SPI1-inducing conditions (open squares: wild-type strain carrying a control plasmid; stars: Δ*hfq* carrying a control plasmid; filled circles: Δ*hfq* complemented with pStHfq-6H).

**Figure S2. The Δ*hfq* mutant is defective for invasion and intracellular replication.**

(A and B) Invasion properties of the Δ*hfq* strain and several control strains. HeLa cells were infected with an MOI of 10, and intracellular bacteria were enumerated 2 hr and 6 hours post infection. (A) Bacteria were grown to early stationary phase in standard LB medium (strains: wild-type, *hfq*^HIS^, *hfq*-C, Δ*hfq*, Δ*spi1,* Δ*rpoS*). (B) Bacteria were grown to late stationary phase under SPI1-inducing conditions (strains: wild-type, *hfq*^HIS^, *hfq*-C, Δ*hfq*, Δ*spi1,* Δ*rpoS*, wild-type strain carrying a control plasmid, Δ*hfq* carrying a control plasmid, Δ*hfq* complemented with pStHfq-6H). (C) RawB macrophages were infected with a MOI of 1 with bacteria grown to early stationary phase (OD of 2), and intracellular bacteria were enumerated 1 hour, 4 hours, and 24 hours post infection. The number of intracellular bacteria is given as percentage of the number of input bacteria. The bacterial strains included were wild-type, *hfq*^HIS^, *hfq*-C, Δ*hfq*,, wild-type strain carrying a control plasmid, Δ*hfq* carrying a control plasmid, Δ*hfq* complemented with pStHfq-6H).

**Figure S3. The Δ*hfq* strain shows an invasion and intracellular growth defect in intestinal epithelial cells and J774A murine macrophage.** (A) Monolayers of LoVo intestinal epithelial cells were infected at a multiplicity of infection (MOI) of 1 or 10 with the wild-type (open circles) and Δ*hfq* strains (filled circles), respectively. One hour post-infection, extracellular bacteria were killed by addition of 50 μg/ml gentamicin and further incubation for one hour. Two hours post-infection, the medium was changed to one containing gentamicin at 10 μg/ml, and infected cells were washed twice with PBS and lysed by addition of 0.1% Triton X-100. Dilutions were plated to agar plates for determination of total intracellular bacteria. Remaining wells were washed and lysed at 4 or 24 hours post-infection. (B) J774A murine macrophages were infected at a MOI of 1 with either the wild-type or Δ*hfq* strains for 30 min, followed by a change of medium containing 50 μg/ml gentamicin for an additional 30 min. The medium was replaced to contain gentamicin at 10 μg/ml, and the first time points (one hour post-infection) were washed and lysed for determination of intracellular bacteria. Remaining samples were taken at 4 and 24 hours post-infection. The results shown are the averages of duplicate wells for each time point, and are representative of at least two, independent experiments.

**Figure S4. The *hfq* mutation leads to various differences in protein levels**

(A) Two dimensional gel electrophoresis of total protein (300 μg) of wt SL1344 and its *hfq* deletion mutant. Protein spots (listed in Table S1) differing in intensity between the two strains were analyzed by MALDI TOF.

**Figure S5. The *hfq* mutant shows reduced adhesion.**

Adherence of *Salmonella* serovar Typhimurium SL1344 and *hfq* mutant strains to HeLa cells. (A) HeLa cells were infected with an MOI of 50. Given is the number of bacteria per HeLa cell one hour post infection, as determined by fluorescence microscopy. (B) Adhesion/invasion assay with bacteria grown to early stationary phase in standard LB medium. Cells were infected for 30 minutes with an MOI of 10 and bacteria enumerated immediately after (no gentamicin-treatment) as well as 2 and 6 hours after infection (with gentamicin-treatment). Open squares: wild-type, filled triangles: *hfq*^HIS^, open diamonds: *hfq*-C, and stars: Δ*hfq*.

**Figure S6. RpoS expression is Hfq-dependent in SL1344.**

(A) Western blot analysis of whole cell protein samples from SL1344 wild-type, Δ*hfq*, and Δ*rpoS* strains. Samples were taken from cultures grown in LB at 37°C at early stationary phase (OD 2). Hybridization was carried out using an *E. coli* RpoS-specific antiserum (Table S2). The quantified RpoS levels are given in relative values of wild-type levels below the lanes.

**Figure S7. Physical maps of plasmids.**

1. pJV300
2. pVP003
3. pVP009 control plasmid
4. pVP012 control plasmid
5. pVP004 complementation plasmid
6. pAS0046 *gfp* transcriptional fusion plasmid
7. pAS0047 *hilA-gfp* transcriptional fusion plasmid
8. pAS0048 *hilA*-5’UTR-*gfp* transcriptional fusion plasmid
9. pAS0057 *ompC-gfp* transcriptional fusion plasmid
10. pAS0058 *ompD-gfp* transcriptional fusion plasmid
11. pVP019 *ompD-gfp* translational fusion plasmid
12. pVP020 *ompC-gfp* translational fusion plasmid

**References**

Chaudhuri, R.R., Khan, A.M., and Pallen, M.J. (2004) coliBASE: an online database for Escherichia coli, Shigella and Salmonella comparative genomics. *Nucleic Acids Res* **32**: D296-299.

Goto, S., Bono, H., Ogata, H., Fujibuchi, W., Nishioka, T., Sato, K., and Kanehisa, M. (1997) Organizing and computing metabolic pathway data in terms of binary relations. *Pac Symp Biocomput*: 175-186.

McClelland, M., Sanderson, K.E., Spieth, J., Clifton, S.W., Latreille, P., Courtney, L., Porwollik, S., Ali, J., Dante, M., Du, F., Hou, S., Layman, D., Leonard, S., Nguyen, C., Scott, K., Holmes, A., Grewal, N., Mulvaney, E., Ryan, E., Sun, H., Florea, L., Miller, W., Stoneking, T., Nhan, M., Waterston, R., and Wilson, R.K. (2001) Complete genome sequence of Salmonella enterica serovar Typhimurium LT2. *Nature* **413**: 852-856.

Zhang, A., Wassarman, K.M., Rosenow, C., Tjaden, B.C., Storz, G., and Gottesman, S. (2003) Global analysis of small RNA and mRNA targets of Hfq. *Mol Microbiol* **50**: 1111-1124.
