# Supplementary figures and images for: The RNA chaperone Hfq is essential for the virulence of Salmonella typhimurium
Source: Mol Microbiol. 2007 Jan;63(1):193–217. doi: 10.1111/j.1365-2958.2006.05489.x (PMC1810395; doi:10.1111/j.1365-2958.2006.05489.x)

## Figure S1

Sittka *et al.*, 2006

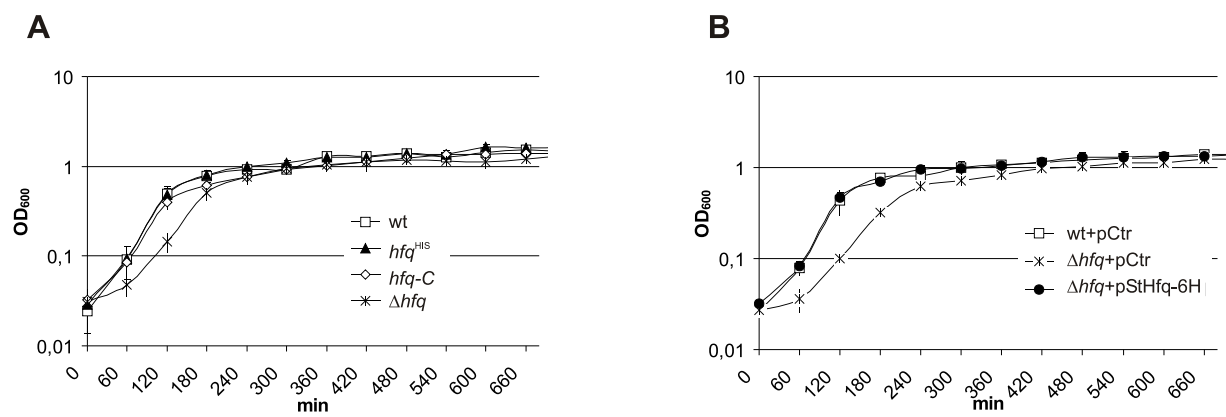

Supplement: Fig. S1 — Growth characteristics of Salmonella strains under SPI1-inducing conditions. [file MMI5489FigS1.pdf]

## Figure S2

Sittka *et al.*, 2006

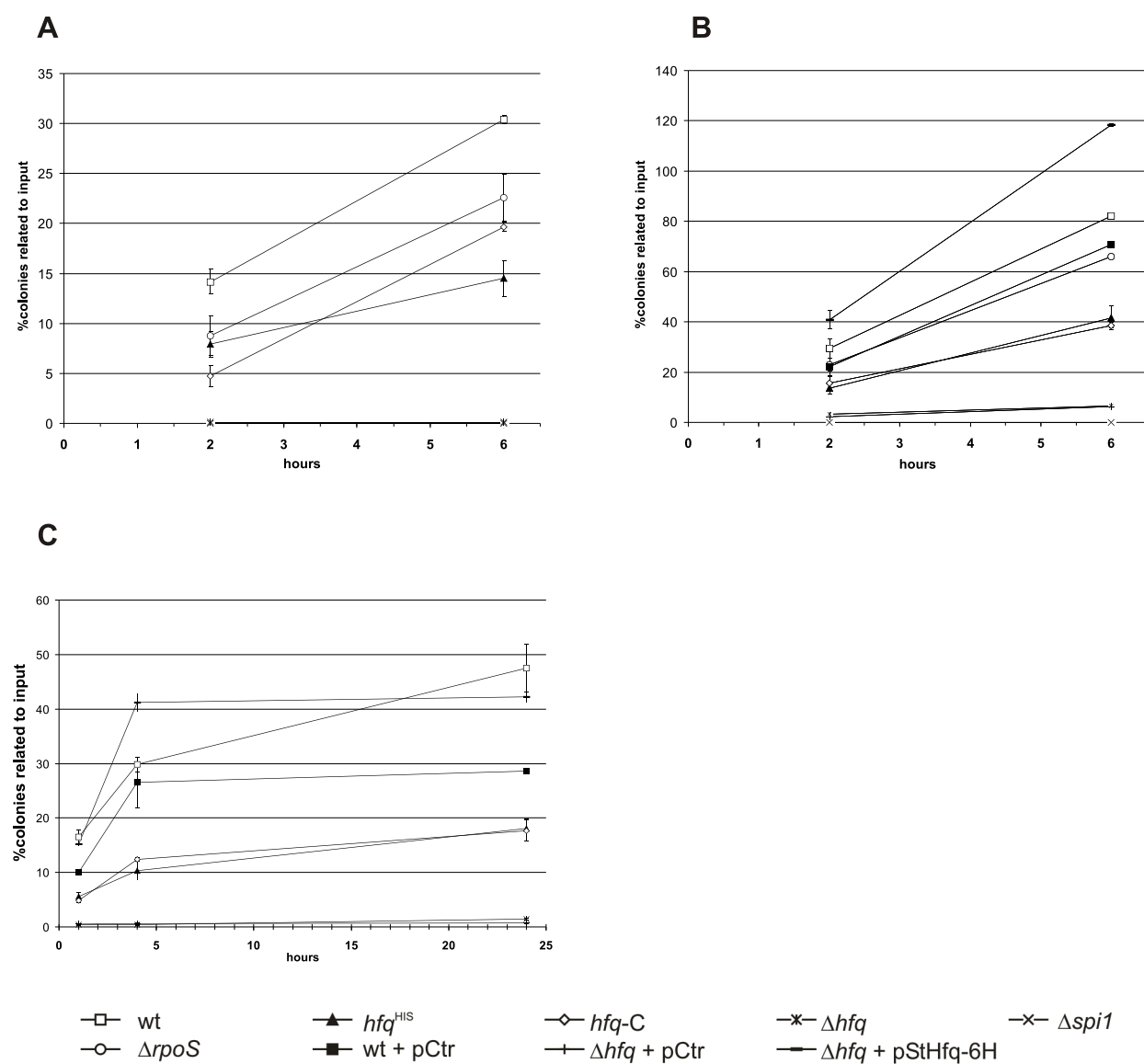

Supplement: Fig. S2 — The Dhfq mutant is defective for invasion and intracellular replication. [file MMI5489FigS2.pdf]

## Figure S3

Sittka *et al.*, 2006

**A**

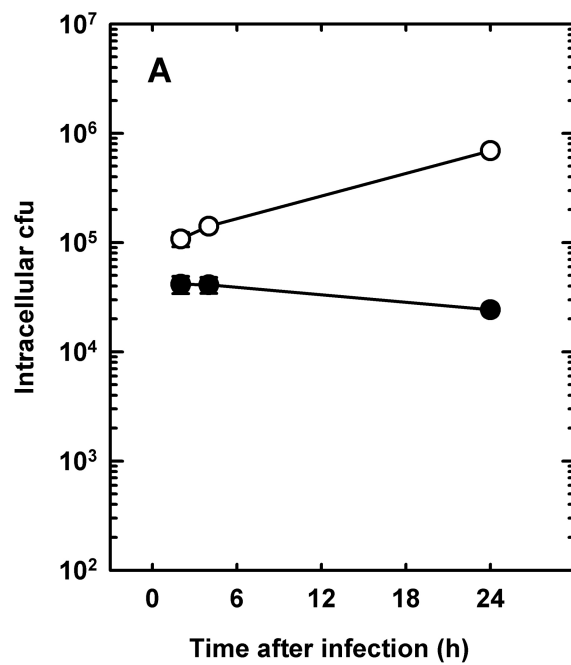

**B**

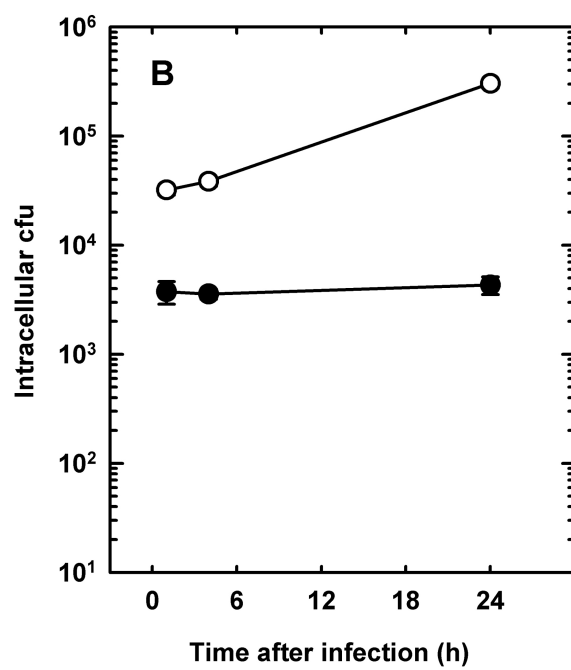

Supplement: Fig. S3 — The Dhfq strain shows an invasion and intracellular growth defect in intestinal epithelial cells and J774A murine macrophage. [file MMI5489FigS3.pdf]

# Figure S4

Sittka *et al.*, 2006

wt

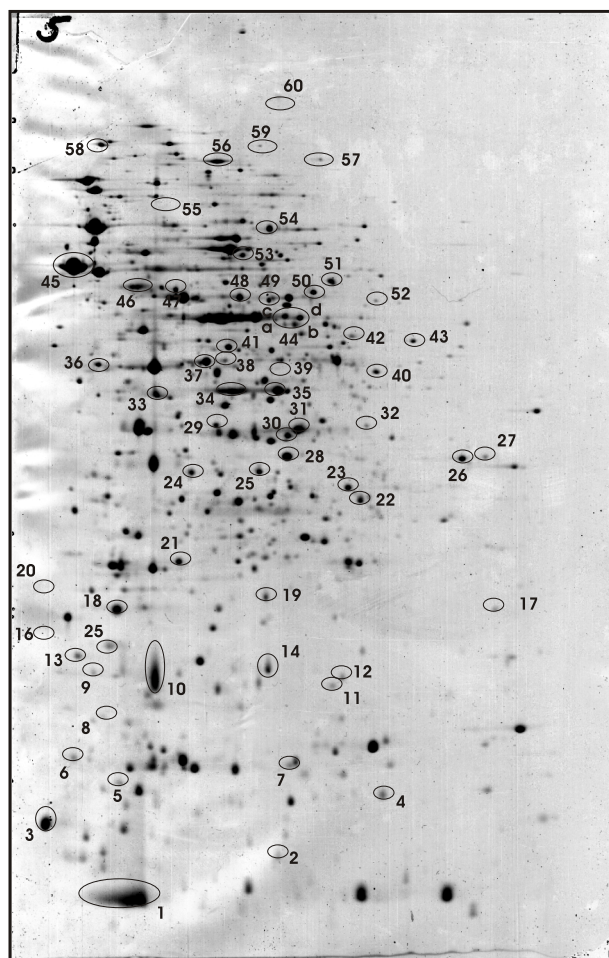

$\Delta hfq$

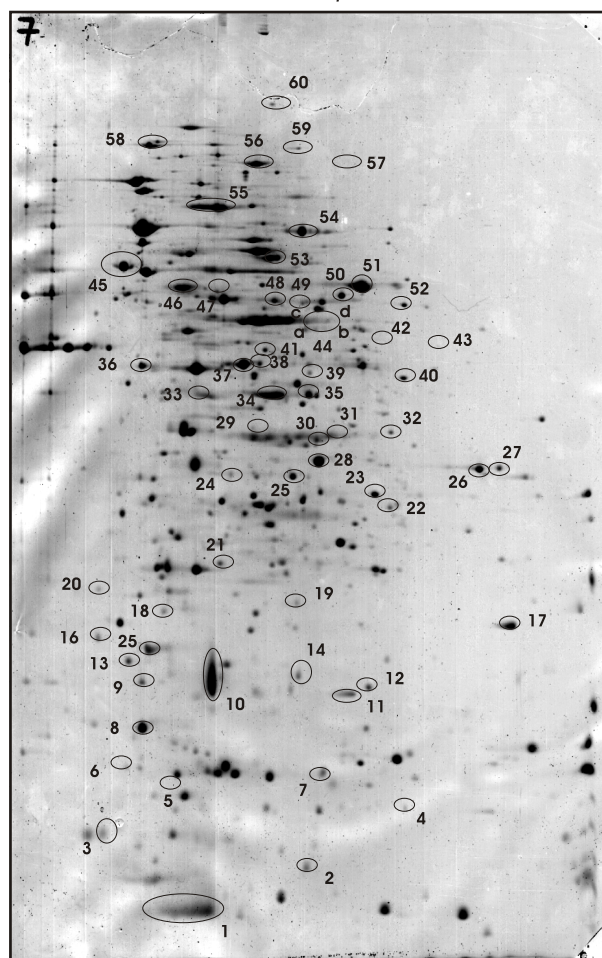

Supplement: Fig. S4 — The hfq mutation leads to various differences in protein levels. [file MMI5489FigS4.pdf]

## Figure S5

Sittka *et al.*, 2006

**A**

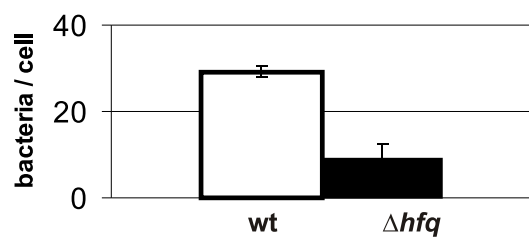

**B**

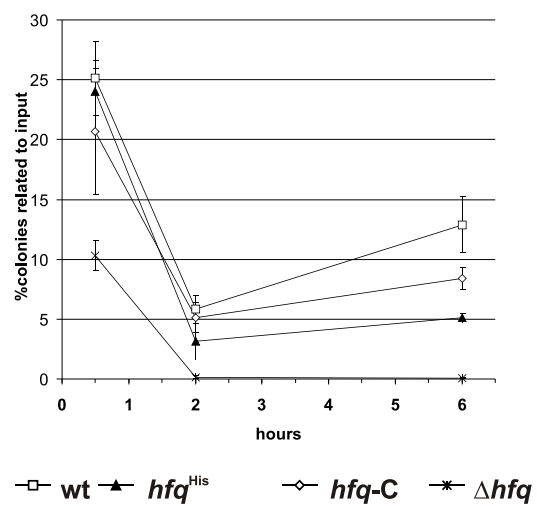

Supplement: Fig. S5 — The hfq mutant shows reduced adhesion. [file MMI5489FigS5.pdf]

## Figure S6

Sittka *et al.*, 2006

**A**

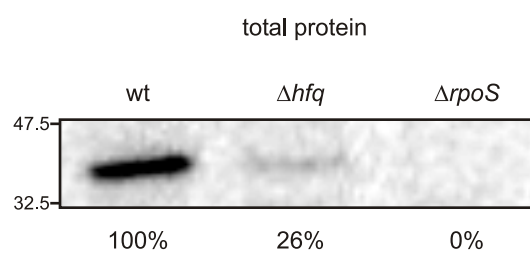

Supplement: Fig. S6 — RpoS expression is Hfq-dependent in SL1344. [file MMI5489FigS6.pdf]

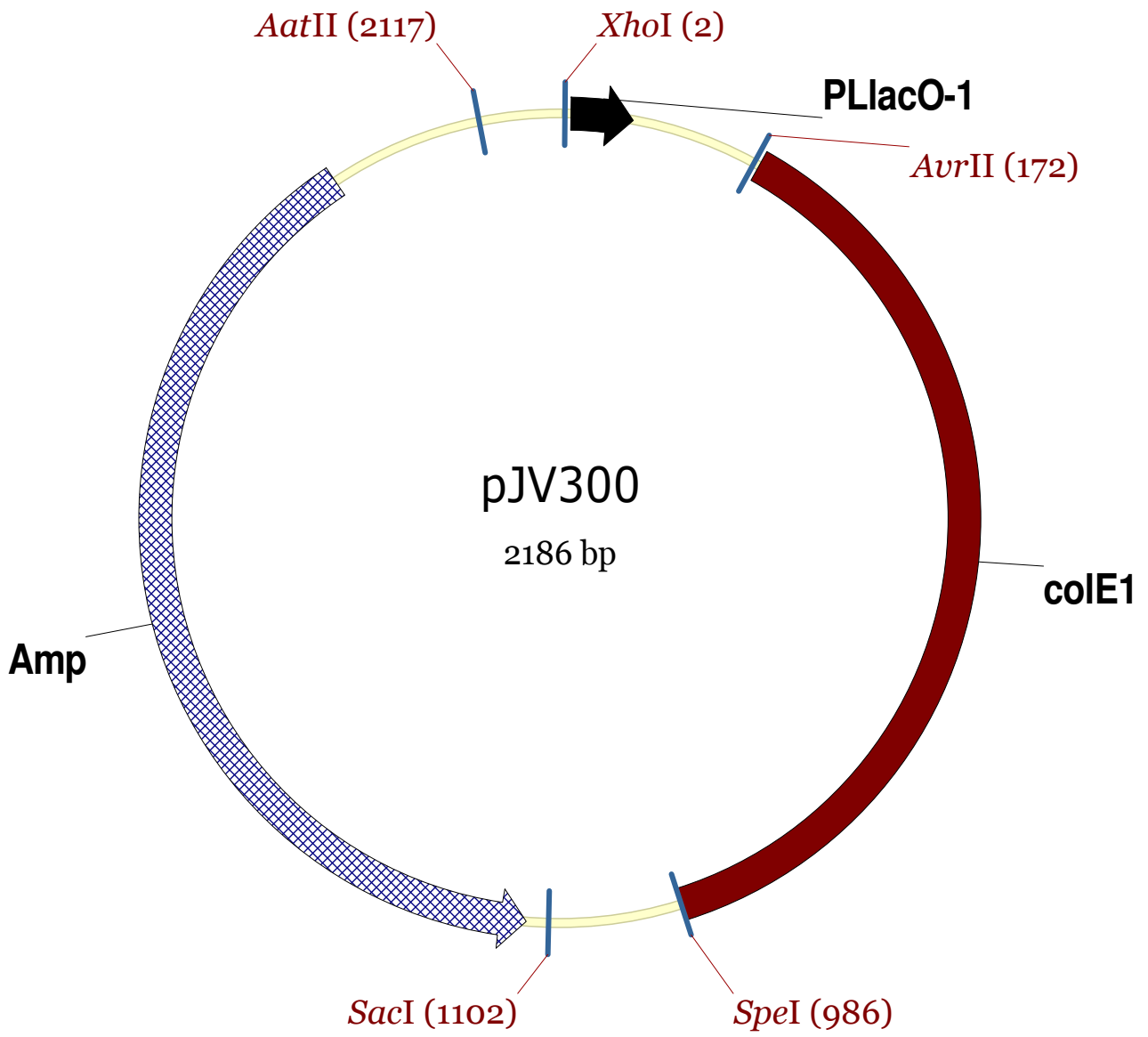

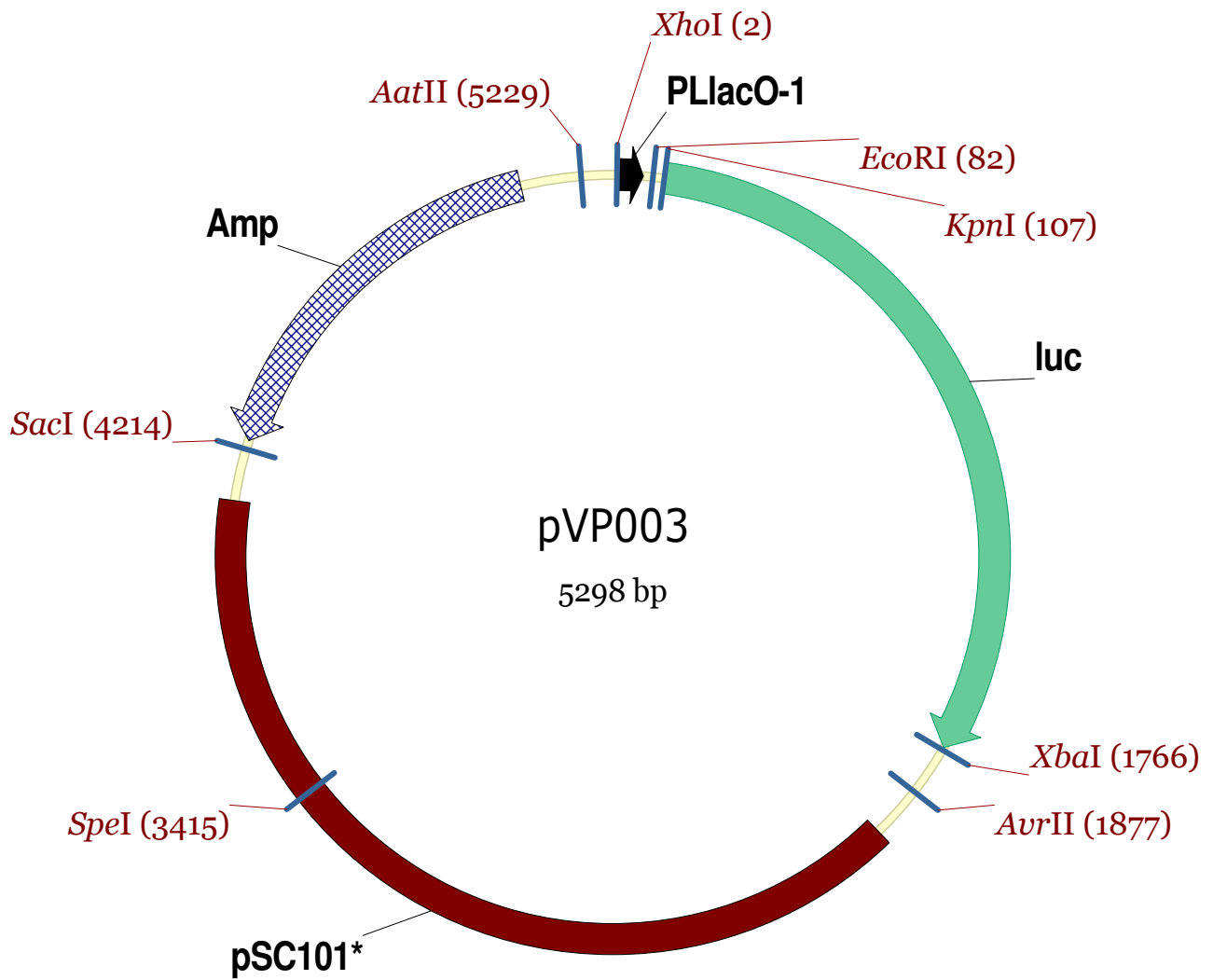

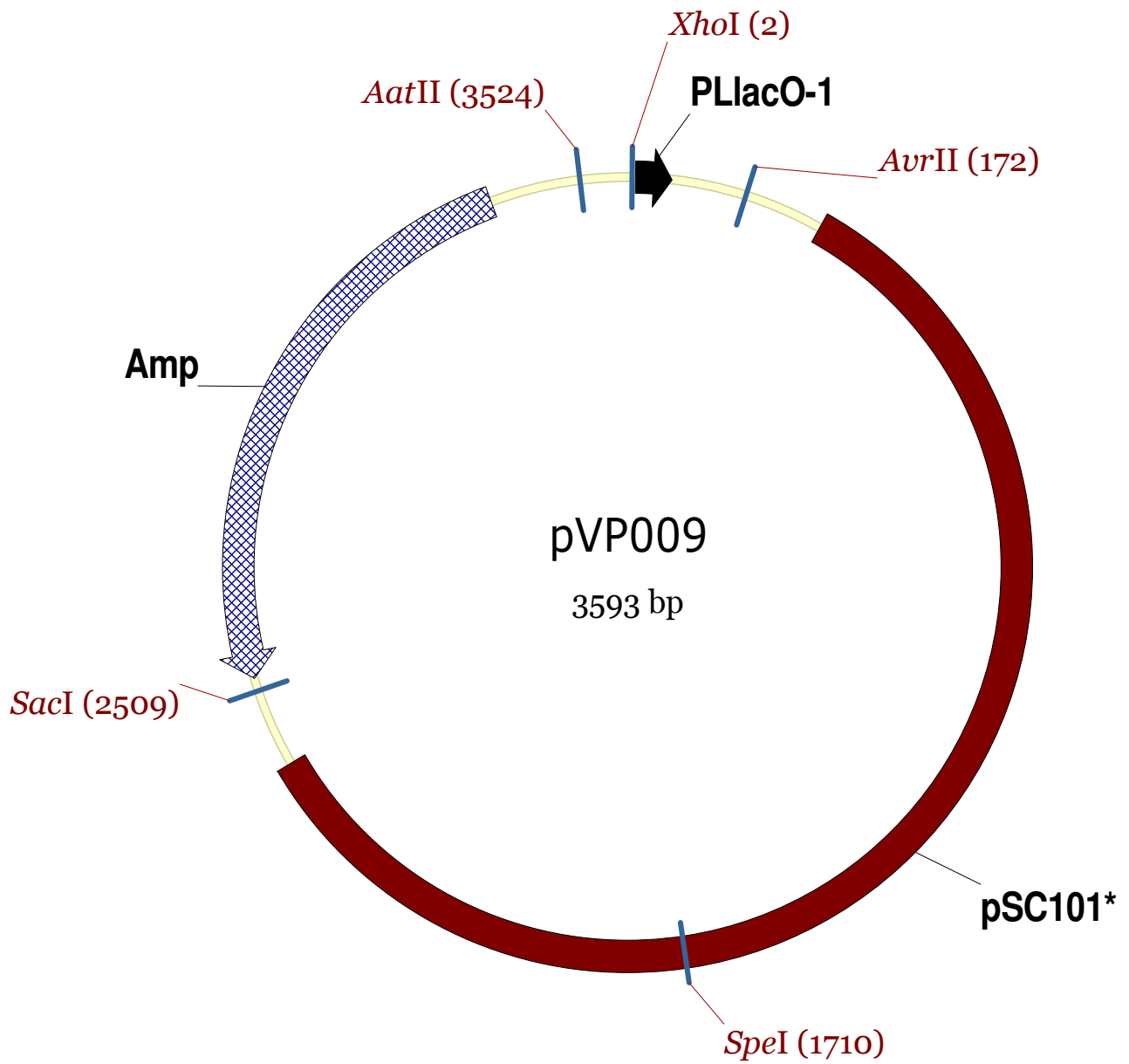

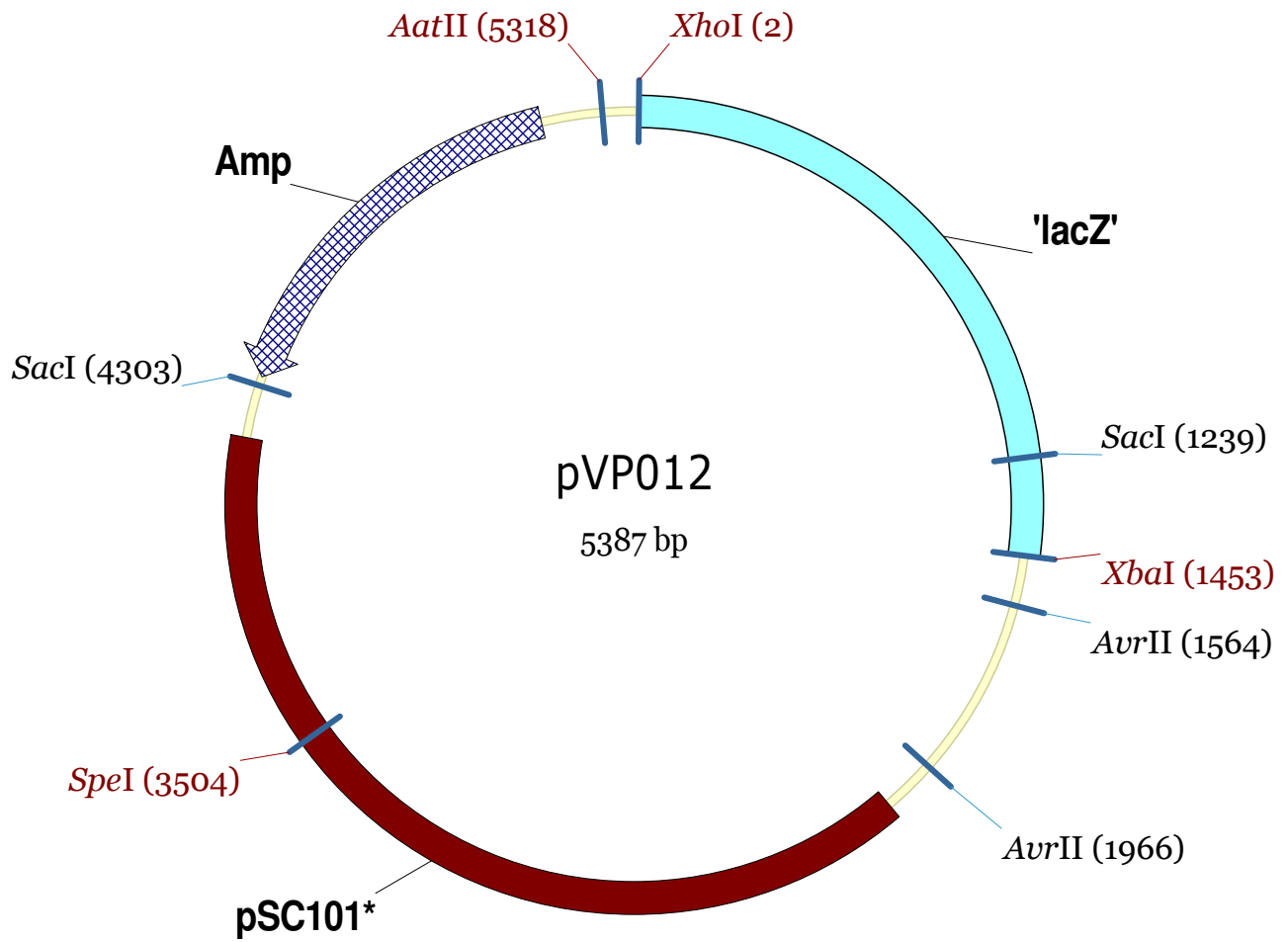

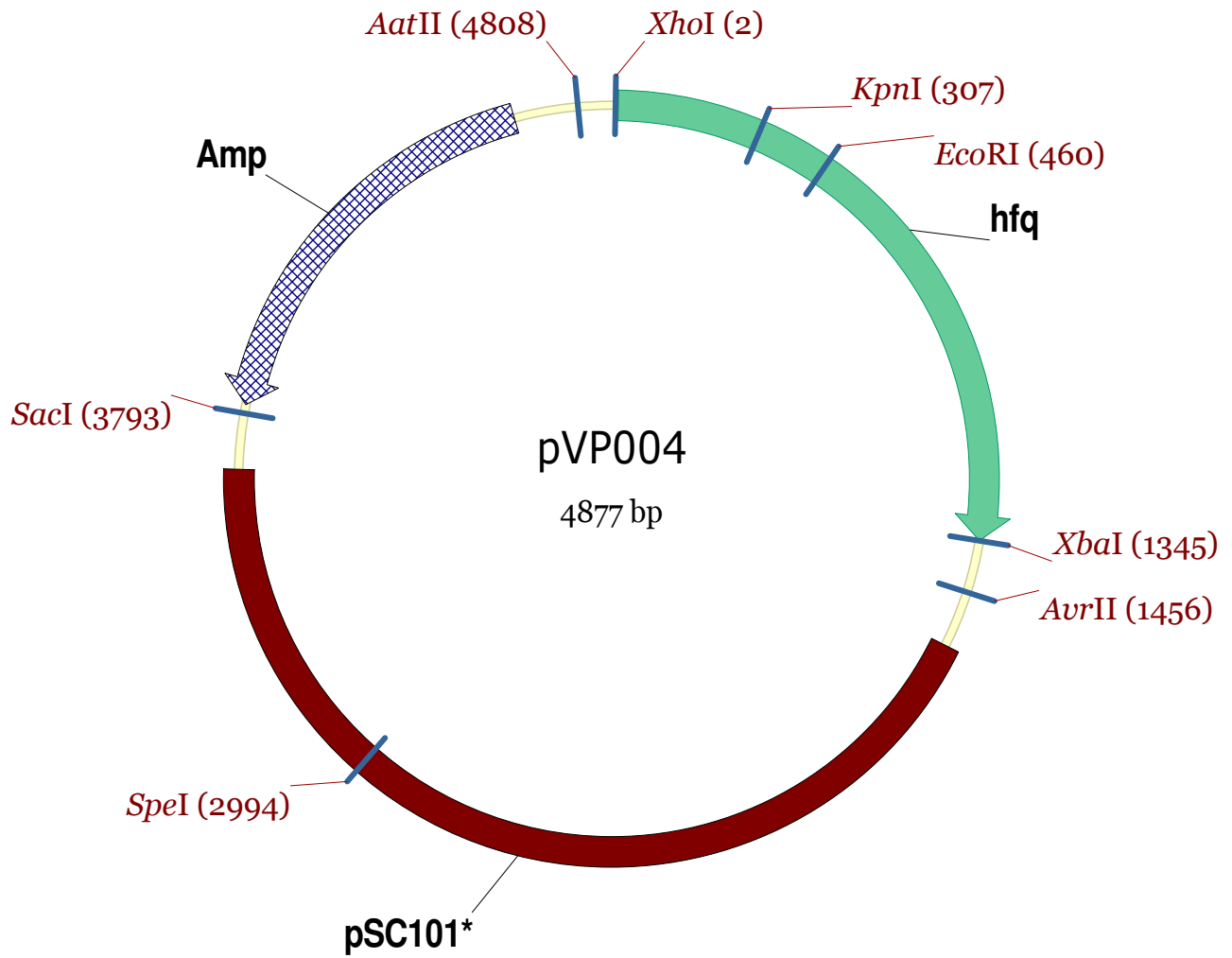

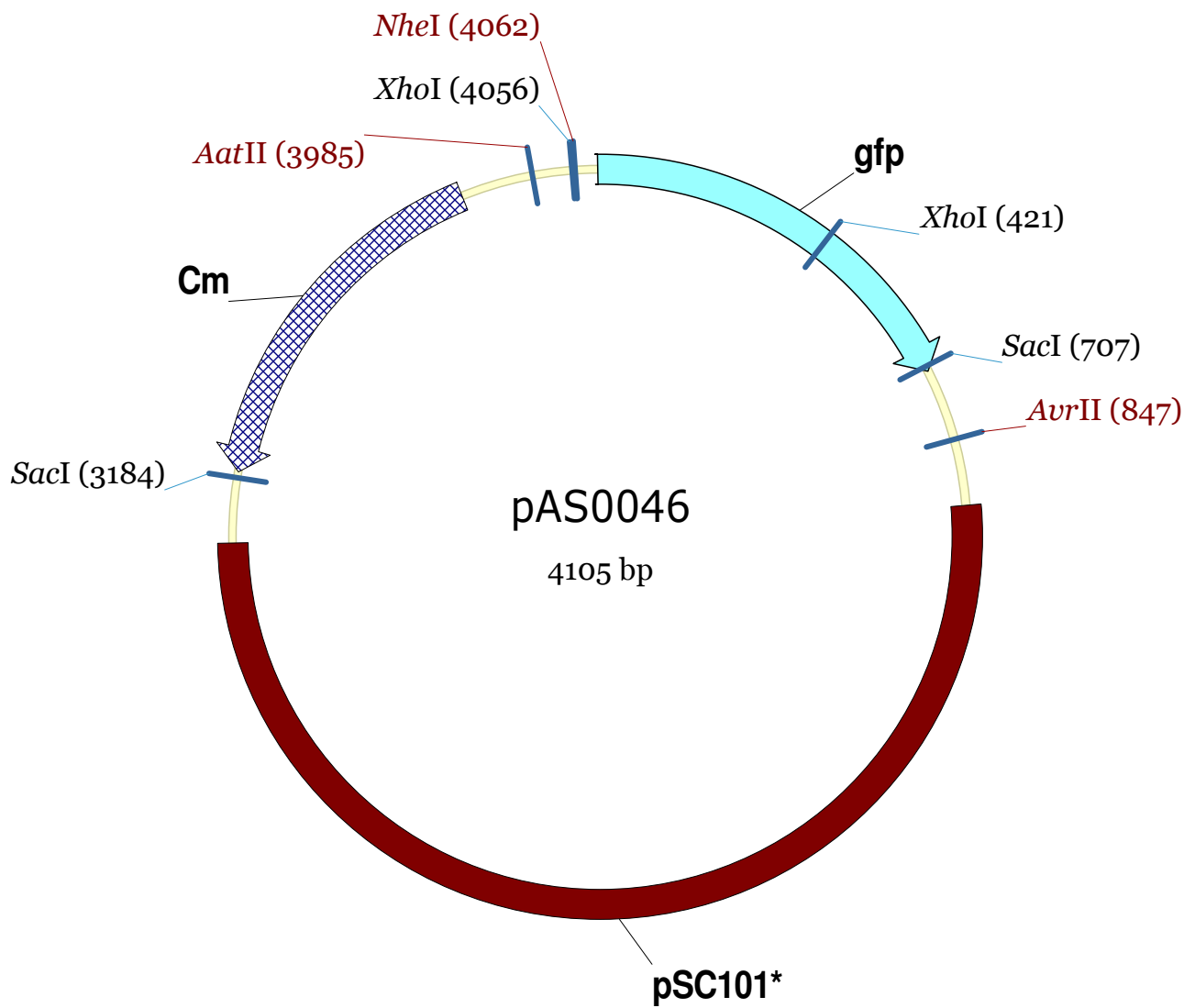

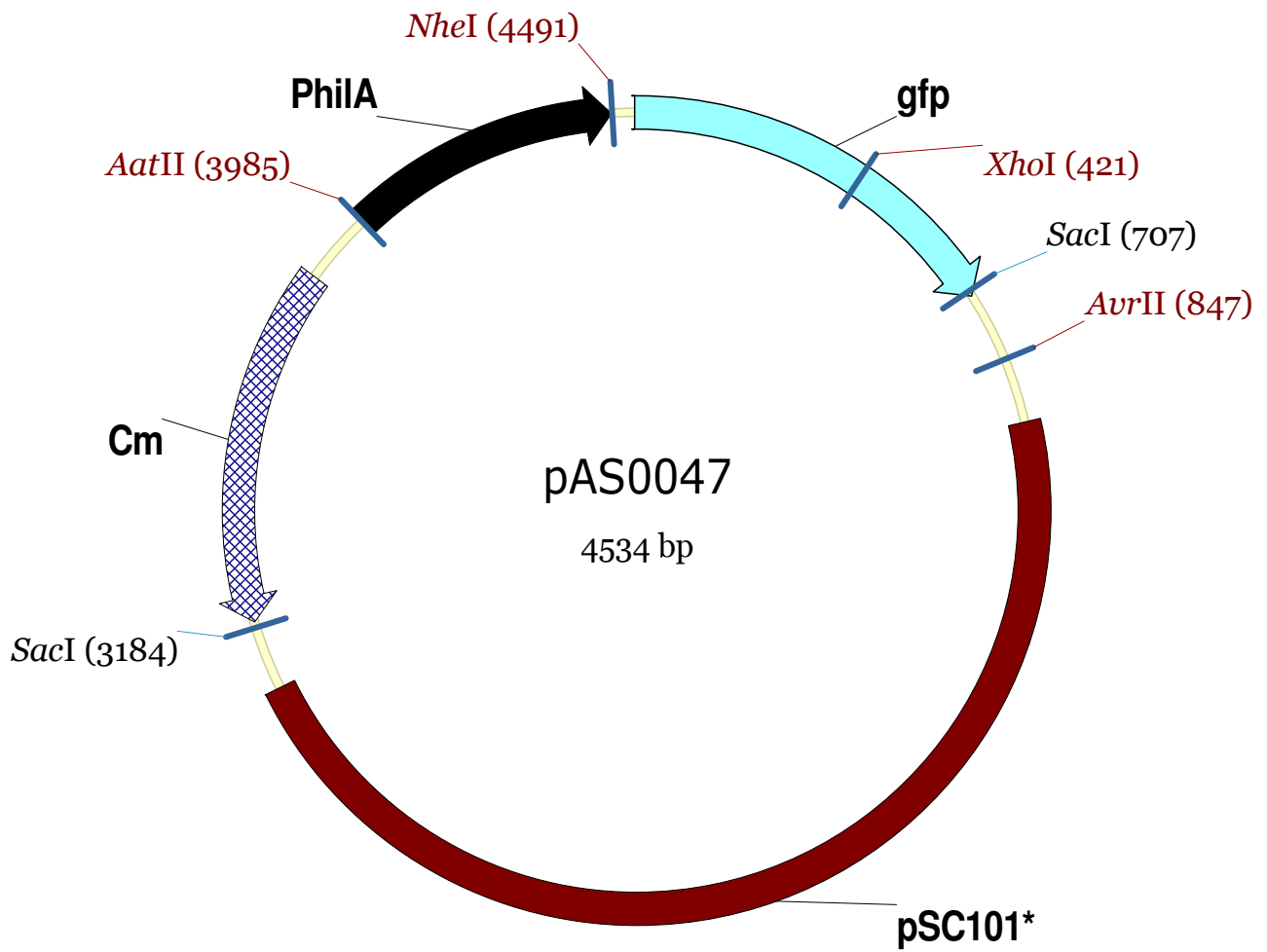

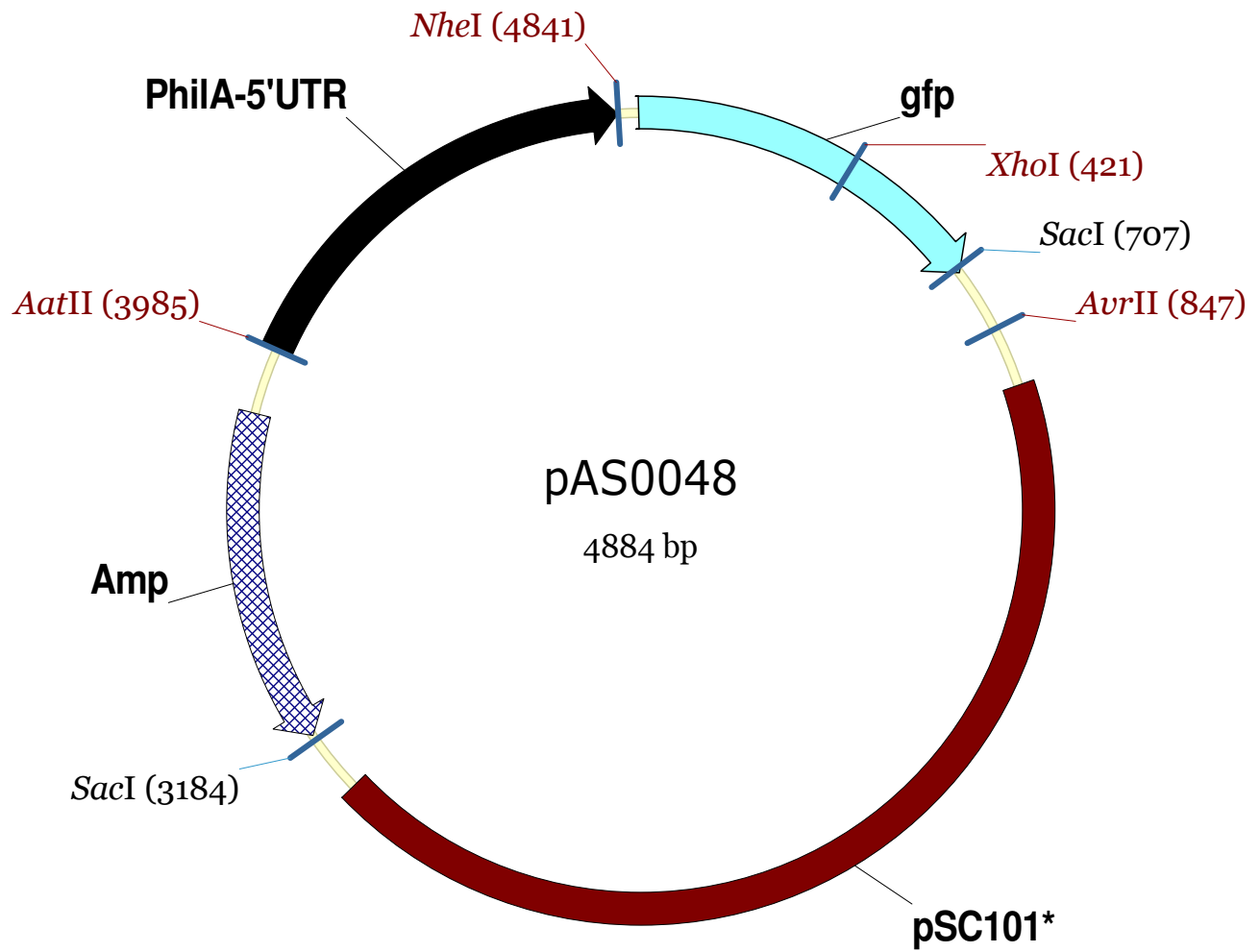

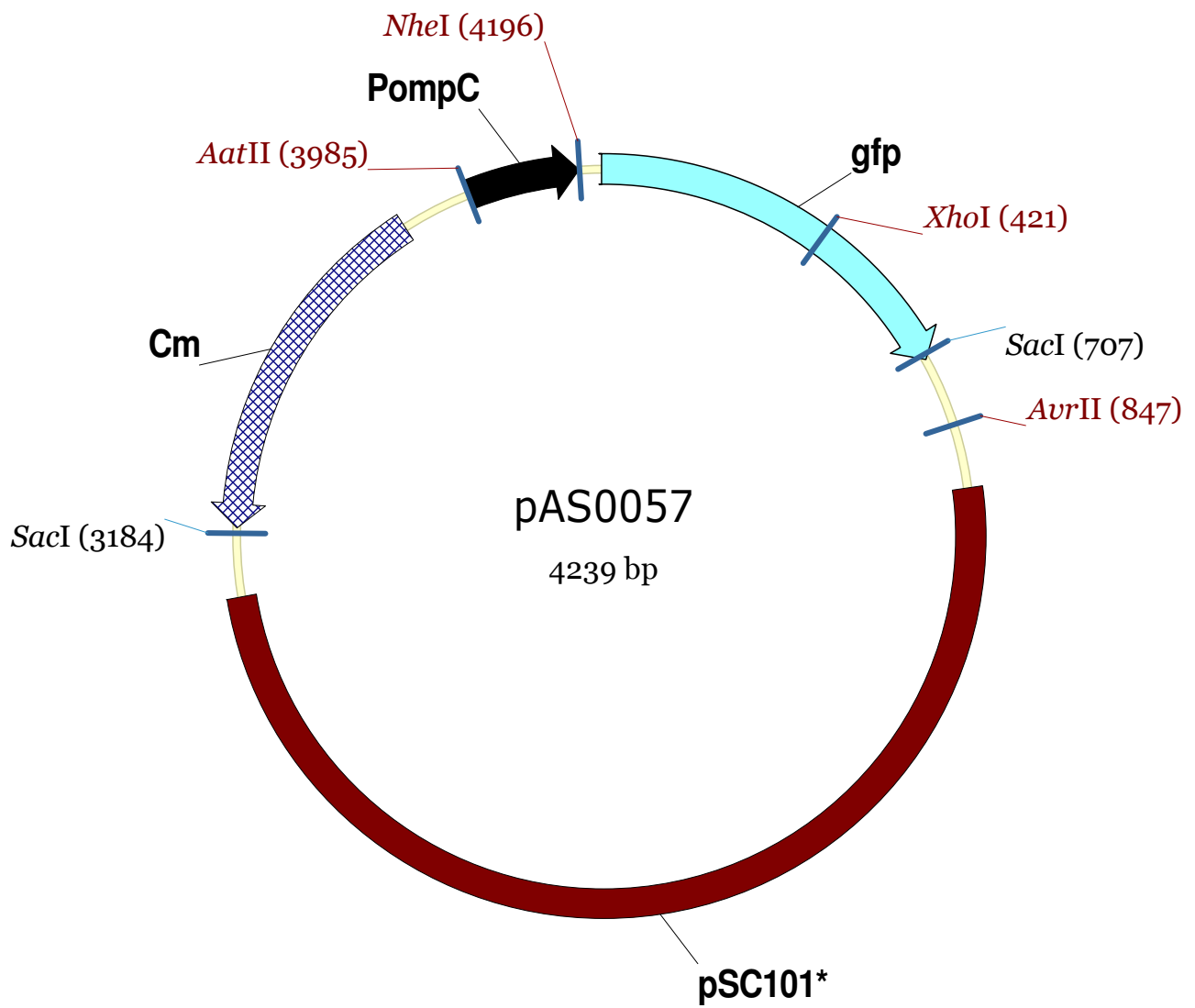

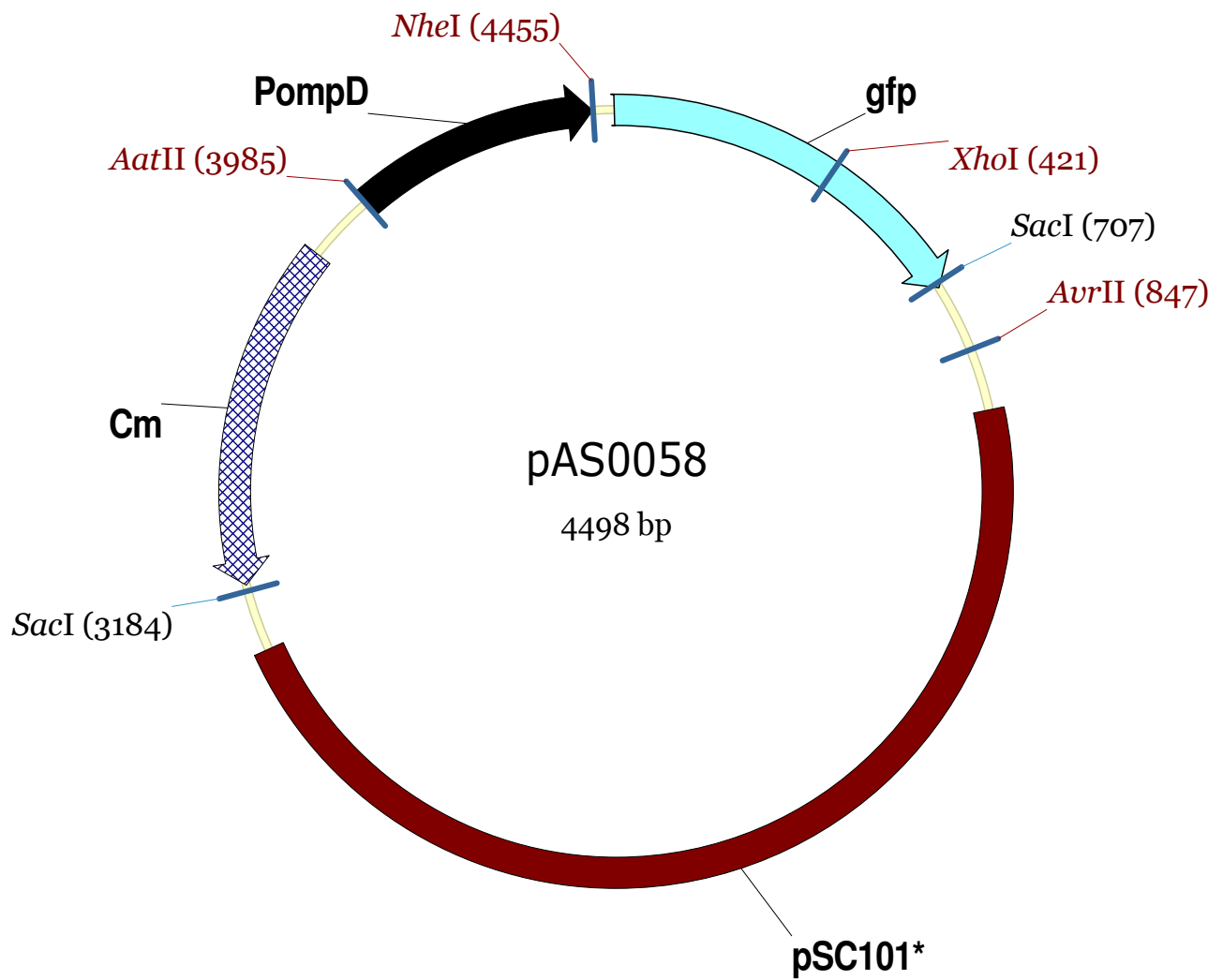

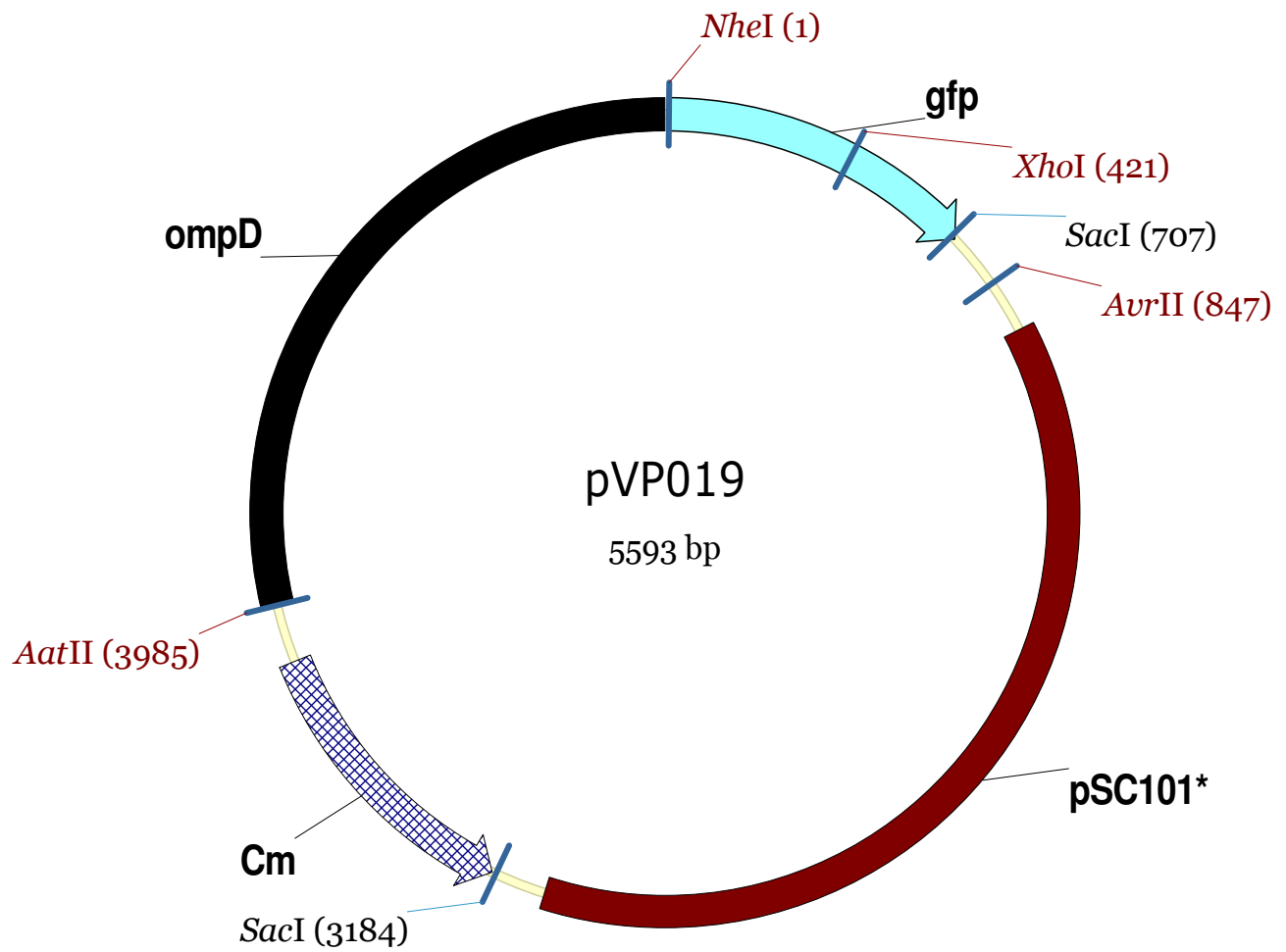

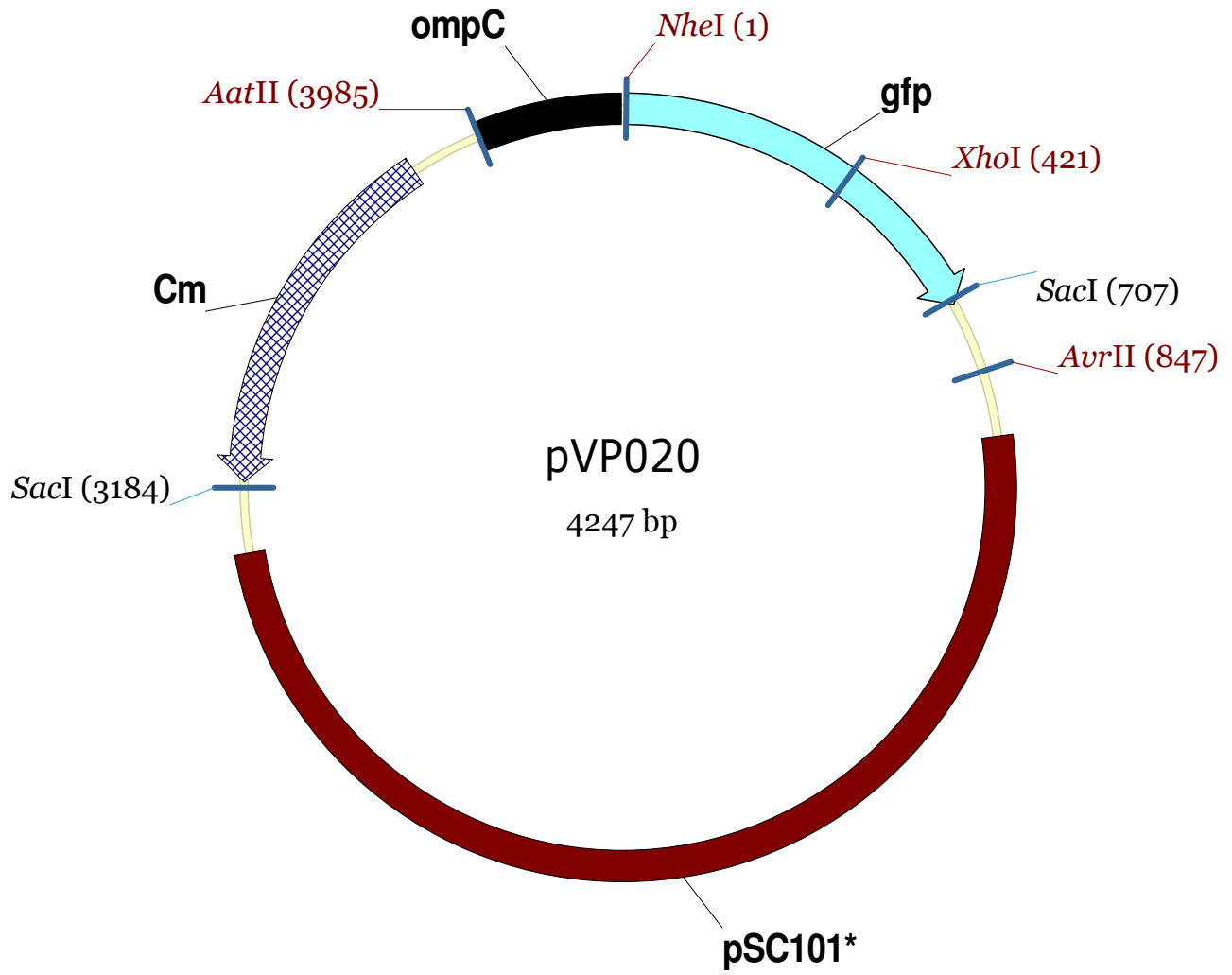

Supplement: Fig. S7 — Physical maps of plasmids. [file MMI5489FigS7.pdf]
